# Penfluridol Triggers GSDME‐Mediated Immunogenic Pyroptosis to Potentiate Antitumor Immunotherapy

**DOI:** 10.1002/advs.76408

**Published:** 2026-07-03

**Authors:** Linfeng Li, Daishi Li, Danyao Chen, Yating Dian, Lei Yao, Hui Su, Ziyu Guo, Deze Zhao, Zihua Wu, Furong Zeng, Chunfang Zhang, Guangtong Deng

**Affiliations:** ^1^ Department of Dermatology Xiangya Hospital Central South University Changsha Hunan China; ^2^ Department of Thoracic Surgery Xiangya Hospital Central South University Changsha Hunan China; ^3^ National Engineering Research Center of Personalized Diagnostic and Therapeutic Technology Changsha Changsha Hunan China; ^4^ Hunan Engineering Research Center of Skin Health and Disease Central South University Changsha Hunan China; ^5^ Hunan Key Laboratory of Skin Cancer and Psoriasis Central South University Changsha Hunan China; ^6^ National Clinical Research Center of Geriatric Disorders Xiangya Hospital Central South University Changsha China; ^7^ Hunan Key Laboratory of Aging Biology Xiangya Hospital Central South University Changsha Hunan China; ^8^ Department of Liver Surgery Xiangya Hospital Central South University Changsha Hunan China; ^9^ Department of Oncology Xiangya Hospital Central South University Changsha Hunan China

**Keywords:** anti‐PD‐1, antipsychotic, GSDME, immunotherapy, PF, pyroptosis

## Abstract

The potential of pyroptosis in antitumor immunity is well‐established; however, its clinical translation is hindered by the lack of safe and effective pyroptosis inducers. Here, using a high‐throughput screen of 240 antipsychotic agents approved by the Food and Drug Administration (FDA), we identify the antipsychotic agent penfluridol (PF) as a potent inducer of pyroptosis via a previously unreported molecular pathway. Mechanistically, PF directly binds to and inhibits TTI1, leading to activation of the TNFA signaling via NFKB and subsequent caspase‐8/caspase‐3‐dependent cleavage of GSDME, culminating in pyroptotic cell death. Preclinically, PF not only exhibits monotherapy efficacy in immunocompetent hosts but also acts synergistically with anti‐PD‐1 therapy in both transplanted and spontaneous melanoma and HCC models without inducing systemic toxicity. Clinically, low TTI1 expression coupled with activated TNFA signaling via NFKB correlates with improved immunotherapy response and prolonged overall survival, suggesting its potential utility as a predictive biomarker. Collectively, our work establishes a compelling paradigm for repurposing pyroptosis inducers to stimulate antitumor immunity.

## Graphical abstract

A high‐throughput screen of FDA‐approved antipsychotics identifies penfluridol as a potent pyroptosis inducer acting via direct TTI1 inhibition. This triggers TNFA‐NFKB signaling and caspase‐8/‐3‐dependent GSDME cleavage. The compound stimulates antitumor immunity alone and synergizes with anti‐PD‐1 therapy, while low TTI1 expression emerges as a potential predictive biomarker for immunotherapy response.

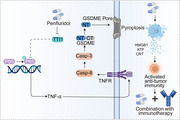

## 1 Introduction

Effective antitumor immunity requires the recognition and elimination of cancer cells by cytotoxic lymphocytes, a process dependent on tumor antigen presentation, co‐stimulatory signals, and inflammatory cues [1, 2]. These elements collectively promote dendritic cell maturation, cytotoxic T cell activation, and the establishment of long‐term immune memory [3, 4]. Critically, the immunogenicity of this process is shaped by the mode of tumor cell death [5]. While immunogenic cell death (ICD) robustly initiates adaptive immunity, conventional apoptosis is often non‐inflammatory or even tolerogenic [6, 7]. Although immune checkpoint blockade can reinvigorate antitumor T cells, its efficacy remains limited in immunologically “cold” tumors, which lack pre‐existing T cell infiltration [8, 9]. Moreover, treatment with immune checkpoint inhibitors is associated with a risk of severe immune‐related adverse events [10], underscoring the need for strategies that specifically enhance antitumor immunity while minimizing systemic toxicity.

Pyroptosis represents a promising mechanism to accomplish this goal. It is a lytic and highly inflammatory type of ICD driven by gasdermin (GSDM) family proteins [11]. Cell swelling, pro‐inflammatory cytokine secretion, and potent immune response amplification are the ultimate consequences of this cell death pathway, which is driven by GSDM cleavage and ensuing plasma membrane pore formation [12, 13]. Among GSDM, GSDME has emerged as a key tumor suppressor [14]. GSDME can be cleaved by caspase‐3—activated upstream by granzyme B from cytotoxic lymphocytes or by initiator caspases such as caspase‐8/9—thereby converting apoptotic signals into potent pyroptosis [15]. This GSDME‐dependent pathway significantly enhances antitumor immunity by amplifying the cytotoxic function of tumor‐infiltrating lymphocytes [16, 17, 18]. Despite compelling evidence supporting the role of GSDM activation in antitumor immunity, the clinical translation of this concept into therapy has been hindered by the scarcity of clinically applicable and safe pyroptosis inducers.

Drug repurposing offers a viable path to rapidly identify such agents, leveraging existing compounds with established safety and pharmacokinetic profiles [19]. Antipsychotic drugs represent particularly attractive candidates due to their blood‐brain barrier penetrance—potentially relevant for treating brain metastases—and their capacity to alleviate cancer‐related neurological symptoms [20, 21]. Penfluridol (PF), a first‐generation diphenylbutylpiperidine antipsychotic, has shown antitumor activity in several cancer types through proposed mechanisms including apoptosis induction and inhibition of oncogenic signaling [22, 23, 24]. However, whether PF can trigger pyroptosis and, more importantly, whether it can remodel the tumor immune microenvironment to synergize with immunotherapy, remains unknown.

Here, through a high‐throughput screen of FDA‐approved antipsychotic compounds, we identify PF as a potent and selective inducer of pyroptosis. We delineate a previously uncharacterized pathway in which PF directly binds and inhibits TTI1, leading to TNFA/NFKB activation, caspase‐8/caspase‐3 cascade signaling, and GSDME cleavage. We demonstrate that PF‐induced pyroptosis is highly immunogenic, initiating a feedforward loop of antitumor immunity that depends on an intact adaptive immune system. Accordingly, PF synergizes with anti‐PD‐1 therapy in multiple murine models, including genetically engineered spontaneous melanoma. Finally, we define a biomarker signature based on low TTI1 expression and high TNFA/NFKB activity that correlates with pyroptosis and predicts improved immunotherapy outcomes in patient cohorts. Our work redefines PF as a novel pyroptosis‐inducing agent and provides a compelling rationale for its repurposing as an immunomodulatory therapy to enhance cancer immunotherapy.

## 2 Results

### 2.1 PF Exhibits Selective Cytotoxicity across Various Cancer Cell Lines

**FIGURE 1:**
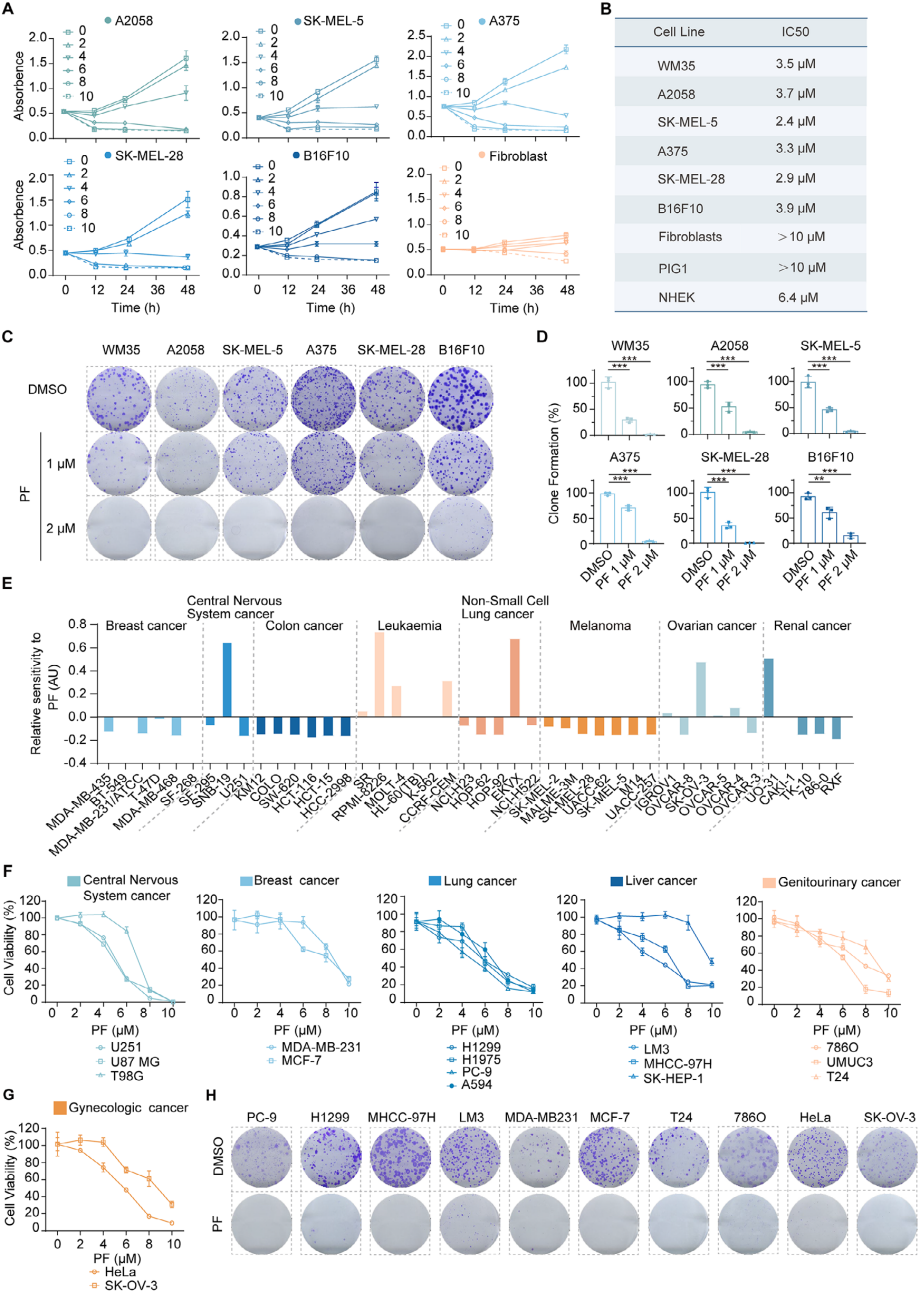
PF exhibits selective cytotoxicity across various cancer cell lines. (A) Quantification of the indicated cell types after treatment with indicated concentration of PF at the indicated time points. (B) The IC50 values of the indicated cell lines following PF treatment. (C, D) Representative images (C) and quantification analysis (D) of clonogenic survival assays in indicated cells following treatment with 1 µM or 2 µM PF for 14 days. (E) Sensitivity profile of 44 cancer cell lines against PF. The cells were grouped based on their cancer types. (F, G) Cell viability of various cancer cell lines (central nervous system, breast, lung, liver, genitourinary, and gynecologic) after treatment with indicated concentrations of PF for 12 h. (H) Representative images of clonogenic survival assays for the indicated cell lines treated with PF for 14 days.

To identify novel anti‐tumor agents, we screened an FDA‐approved library of 240 antipsychotic compounds in the human melanoma cell line A375. PF exhibited the most potent anti‐cancer activity (Figure S1A and Table S1). PF also induced time‐dependent and dose‐dependent growth inhibition and cell death in a panel of melanoma cell lines, but not in human fibroblast cells, human melanocyte cell PIG1, and human keratinocyte cell NHEK (Figure 1A,B). Further evidence from colony formation assays showed that PF was capable of completely suppressing the long‐term growth of the tumor cells (Figure 1C,D). The anti‐cancer potency of PF was also independently evaluated using the NCI‐60 cancer cell line panel established by the United States National Cancer Institute [25]. The NCI‐60 screen indicated heightened sensitivity in breast cancer, colon cancer, and melanoma cells compared to other cancer types (Figure 1E). We further validated the anti‐cancer potency of PF in breast cancer, and also observed that PF inhibited the cell viability of central nervous system cancer, lung cancer, liver cancer, genitourinary tumors, and gynecologic tumor cells in a dose‐dependent manner (Figure 1F,G). Colony formation assays further confirmed sustained growth inhibition across these cancer types (Figure 1H). Together, these findings suggest that PF selectively inhibits growth and induces cell death in tumor cells, with minimal impact on normal cells.

### 2.2 PF Triggers GSDME‐Mediated Pyroptosis through Inducing Caspase 8/3 Cleavage

**FIGURE 2:**
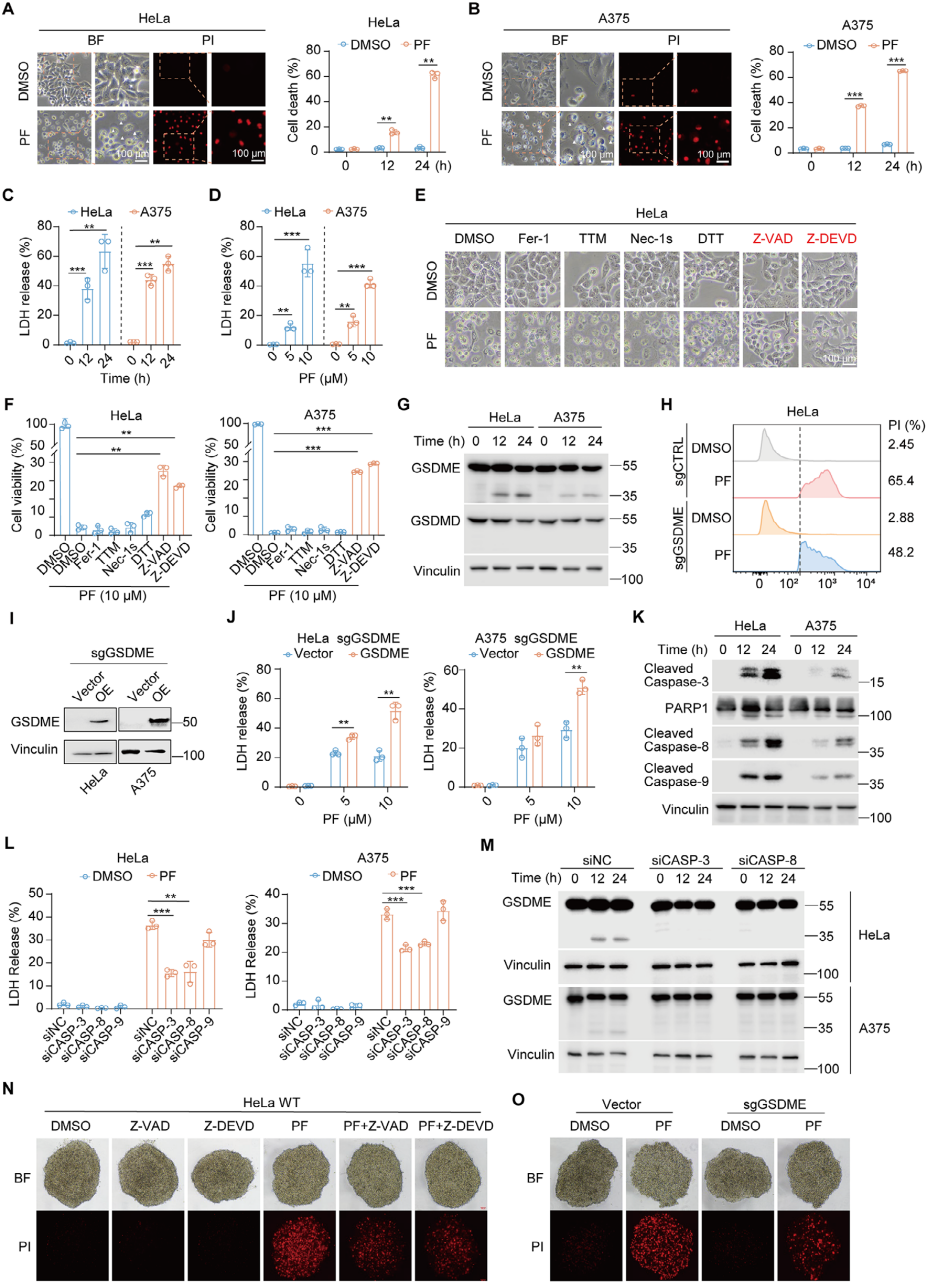
PF triggers GSDME‐mediated pyroptosis through induing caspase 8/3 cleavage. (A, B) Representative images of bright field (BF), PI staining, and corresponding flow cytometry analysis of cell death in HeLa (A) and A375 (B) cells following treatment with 10 µm PF at the indicated time points. (C, D) Measurement of LDH release in response to PF treatment; (C) Cells were treated with 10 µm PF for the indicated time points; (D) Cells were treated with the indicated concentrations of PF for 10 h. (E, F) Representative images of HeLa cells (E) and quantification of cell viability in HeLa and A375 cells (F) following treatment with PF and various inhibitors: 5 µm Fer‐1; 10 µm TTM; 10 µm Nec‐1s; 10 µm DTT; 10 µm CQ; 40 µm Z‐DEVD; 40 µm Z‐VAD; G) Western blot analysis of cleavage of GSDME and GSDMD in HeLa and A375 cells following treatment with 10 µm PF for the indicated times. (H) Representative histograms of cell death in sgCTRL and sgGSDME HeLa and A375 cells treated with 10 µm PF for 10 h. (I) Western blot analysis confirms GSDME knockout and successful reconstitution of GSDME expression in HeLa and A375 cells. (J) LDH release was assessed in sgGSDME and OEGSDME cells following treatment with the indicated concentrations of PF for 10 h. (K) The expression of cleaved caspase‐3, PARP1, cleaved caspase‐8, and cleaved caspase‐9 was analyzed by western blot after treatment with 10 µm PF for the indicated times. (L) Measurement of LDH release in siNC, siCASPASE3, or siCASPASE8, and siCASPASE9‐transfected HeLa and A375 cells following treatment with 10 µm PF for 10 h. (M) Western blot analysis of GSDME cleavage in HeLa and A375 cells transfected with siNC, siCASPASE‐3, or siCASPASE‐8, following treatment with 10 µm PF for the indicated times. (N) Representative images of 3D HeLa tumor spheroids following treatment with 40 µm Z‐VAD or 40 µm Z‐DEVD, in the presence or absence of 10 µm PF for 10 h. (O) Representative images of 3D HeLa tumor spheroids in sgCTRL and sgGSDME cells following treatment with 10 µm PF for 10 h.

To determine whether PF induces known forms of regulated cell death, we first assessed cellular morphology changes in PF‐treated A375 and HeLa cells. We observed classic features of pyroptosis, including loss of membrane integrity, cell rounding, and swelling (Figure 2A,B; Figure S2A,B) [11]. Propidium iodide (PI) staining further confirmed that PF‐induced cell death was associated with compromised membrane integrity (Figure 2A,B). Additionally, lactate dehydrogenase (LDH) detection assay revealed that PF facilitated LDH release (Figure 2C,D; Figure S2C,D), indicating lytic cell death. These findings supported that the anticancer activity of PF depends on the induction of pyroptosis.

Pyroptosis is molecularly executed by members of the GSDM family following their proteolytic cleavage into active N‐terminal fragments by specific caspases [26]. The cytotoxicity of PF was largely abolished by pre‐treatment with Z‐VAD‐FMK (Z‐VAD, a pan‐caspase inhibitor) or Z‐DEVD‐FMK (Z‐DEVD, a caspase‐3/8 inhibitor), but not by inhibitors of necroptosis, disulfidptosis, cuproptosis, or ferroptosis (Figure 2E,F; Figure S2E). Moreover, PF specifically induced the cleavage of GSDME, but not GSDMD (Figure 2G). Genetic ablation of GSDME significantly attenuated PF‐induced cytotoxicity (Figure 2H; Figure S2F,G), whereas reintroduction of GSDME in GSDME‐deficient cells restored sensitivity to PF (Figure 2I,J). These results indicate that PF induces pyroptosis via caspase‐3‐dependent cleavage of GSDME. In line with these findings, PF treatment increased the levels of cleaved caspase‐3, cleaved PARP1, cleaved caspase‐8, and cleaved caspase‐9 (Figure 2K), but did not increase cleaved caspase‐1, an upstream activator of GSDMD [27] (Figure S2H). Knockdown of caspase‐9 did not block PF‐induced cell death, whereas depletion of either caspase‐3 or caspase‐8 inhibited cell death and LDH release (Figure 2L; Figure S2I–K). These results were consistent with the previous findings that caspase‐8 cleaves and activates caspase‐3, which then cleaves GSDME [28]. Notably, although GSDME knockout or Caspase knockdown strongly inhibited PF‐induced LDH release—a hallmark of lytic cell death—the recovery of overall cell viability was only partial (Figure 2L; Figure S2K). This dissociation indicates that while GSDME‐dependent pyroptosis primarily drives membrane rupture upon PF exposure, it does not fully account for the overall cytotoxic effect. The residual loss of viability after pyroptosis blockade suggests the involvement of other regulated cell death pathways, such as apoptosis and autophagy, which impair metabolic function without immediate lysis, consistent with previous findings [29, 30, 31, 32, 33]. Furthermore, caspase‐3/8 knockdown attenuates PF‑induced GSDME cleavage, confirming caspase‑3/8‑dependent pyroptosis (Figure 2M). With the aim of confirming these results using a model of higher physiological relevance, we established 3D tumor spheroids and found that PF‐induced pyroptosis could be almost completely rescued by Z‐VAD, Z‐DEVD, or GSDME knockout (Figure 2N,O). Collectively, these findings demonstrate that PF triggers GSDME‐mediated pyroptosis through sequential activation of caspase‐8 and caspase‐3.

### 2.3 TTI1 Inhibition Activates TNFA Signaling via NFKB and Contributes to PF‐Induced Pyroptosis

**FIGURE 3:**
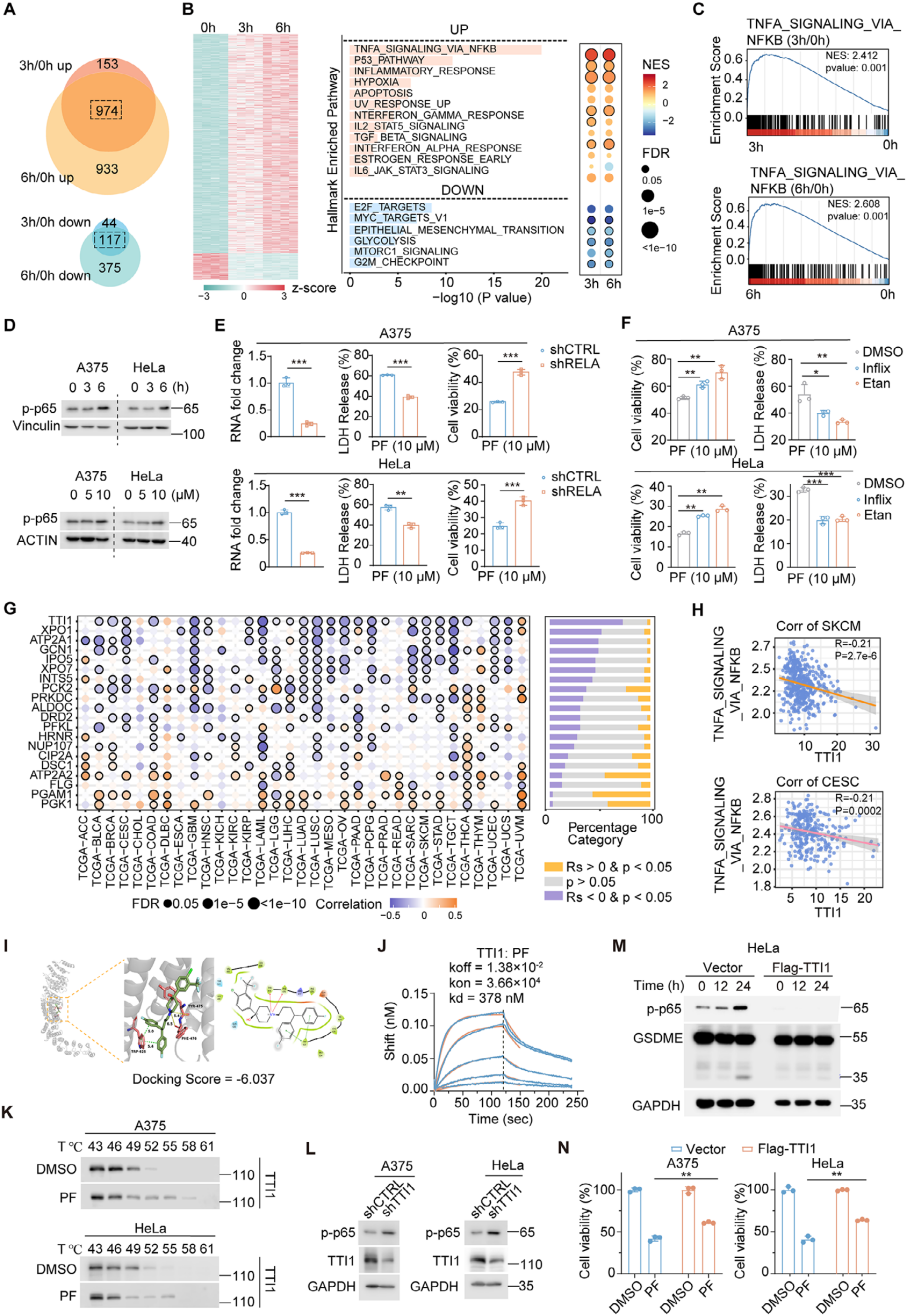
TTI1 inhibition activates TNFA signaling via NFKB and contributes to PF‐induced pyroptosis. (A) Venn diagram shows the common differentially expressed up‐regulated genes (top) and down‐regulated genes (bottom) of PF treatment for 3 h and 6 h. (B) Heatmap of differentially expressed genes (DEGs) following PF treatment at 3 h and 6 h versus untreated control (left), significant enrichment of Hallmark pathways among the up and down regulated differentially expressed genes (right). (C) GSEA revealed significant enrichment of the “TNFA Signaling Via NFKB” pathway following both 3‐h (top) and 6‐h (bottom) PF treatments. (D) The expression of p‐p65 was determined by western blot after treatment with 10 µm PF for the indicated times. (E) Measurement of LDH release and cell viability in shCTRL and shRELA HeLa and A375 cells after treatment with 10 µm PF for 10 h. (F) Measurement of LDH release and cell viability in HeLa and A375 cells following treatment with 10 µm PF, 100 ng/ml infliximab or etanercept. (G) The correlation between potential PF targets and “TNFA Signaling Via NFKB pathway” in different tumors within the TCGA database. (H) Scatter plot depicting the correlation between TTI1 expression and TNFA pathway activity in SKCM and CESC. (I) Molecular‐docking showing the binding between PF and TTI1. (J) Bio‐layer interferometry (BLI) assay of PF binding to recombinant TTI1 protein. (K) The effects of PF on thermal stability of the TTI1 protein in A375 and HeLa cells. (L) Western blot analysis of p‐p65 levels and TTI1 expression in HeLa and A375 cells expressing shNC or shTTI1. (M) Western blot analysis of p‐p65 levels and GSDME cleavage in HeLa Vector and OETTI1 cells following exposure to 10 µM PF for the indicated time. (N) Measurement of cell viability in Vector and OETTI1 A375 and HeLa cells following treatment with 10 µM PF.

To further clarify the underlying mechanism of PF‐induced pyroptosis, we performed high‐throughput RNA sequencing in A375 cells treated with PF for 0, 3, and 6 h, respectively. Principal component analysis (PCA) showed progressive changes and could be distinguished into three groups (Figure S3A). PF treatment led to significant transcriptomic changes, with 974 genes upregulated and 117 downregulated after both 3 h and 6 h treatments (Figure 3A; Figure S3B). Hallmark pathway analysis revealed pronounced activation of multiple inflammatory response pathways in the differentially expressed genes, with TNFA signaling via NFKB being among the most strongly enriched (Figure 3B). Gene Set Enrichment Analysis (GSEA) further confirmed that PF robustly activates TNFA signaling via NFKB (Figure 3C). Consistently, we observed increased NFKB transcriptional activity in PF‐treated cells, as evidenced by elevated phosphorylation of p65 (Figure 3D) [34]. To determine the pivotal role of TNFA signaling via NFKB, we found that RELA (which encodes p65) knockdown in these tumor cells reversed PF‐induced cell death and LDH release (Figure 3E). Moreover, treatment with TNFA inhibitors, infliximab and etanercept consistently attenuated PF‐induced cell death and LDH release (Figure 3F). These results suggested that activation of TNFA signaling via NFKB contributes to PF‐induced pyroptosis.

PF has been reported to directly target a series of proteins in cancer cells [30, 32]. To identify which target might link PF to TNFA signaling via NFKB, we analyzed the correlation between the expression of known PF targets and the activity of TNFA signaling via NFKB using TCGA data. TTI1 emerged as one of the most negatively correlated targets (Figure 3G), a finding further validated in the CCLE database (Figure 3H). Molecular docking analyses suggested a potential interaction between PF and TTI1, evidenced by a docking score of −6.073 kcal mol^−^^1^ (Figure 3I). Bio‐layer interferometry (BLI) revealed a direct binding of PF to full‑length TTI1. The determined Kd value of 378 nM reflects a potent and specific molecular interaction (Figure 3J). Cellular thermal shift assay (CETSA) showed that TTI1 exhibits improved thermal stability in A375 and HeLa cells after PF treatment (Figure 3K). Having established direct binding between PF and TTI1, we next examined the functional link between TTI1 and NFKB signaling. TTI1 knockdown activated p‐p65, indicating that TTI1 negatively regulates this pathway (Figure 3L). Moreover, TTI1 knockdown sensitized tumor cells to PF‑mediated cytotoxicity (Figure S3C–E). In contrast, modulation of other reported targets, such as CIP2A and PFKL, did not affect PF sensitivity, indicating a specific role for TTI1 (Figure S3F–I). To clarify whether TTI1 mediates PF‐induced pyroptosis specifically through TNFA/NFKB signaling, we constructed TTI1‐overexpressing cell lines (Figure S3J) and found that overexpression of TTI1 inhibited the activation of p‐p65 and the cleavage of GSDME following PF treatment, and significantly reversed PF‐induced cell death (Figure 3M,N; Figure S3K). We further examined the expression of TTI1 and GSDME across a panel of normal and tumor cell lines and found that TTI1, but not GSDME, was specifically upregulated in tumor cells (Figure S3L). Moreover, data from The Human Protein Atlas revealed that TTI1 is also specifically upregulated in tumor tissues compared with adjacent normal tissues (Figure S3M). These findings provide a possible explanation for the selective cytotoxicity of PF toward tumor cells. Altogether, these results support a model where PF binds to and inhibits TTI1, leading to activation of TNFA/NFKB signaling and subsequent pyroptosis.

### 2.4 PF Trigger Immunogenic Pyroptosis to Activate Anti‐Tumor Immunity

**FIGURE 4:**
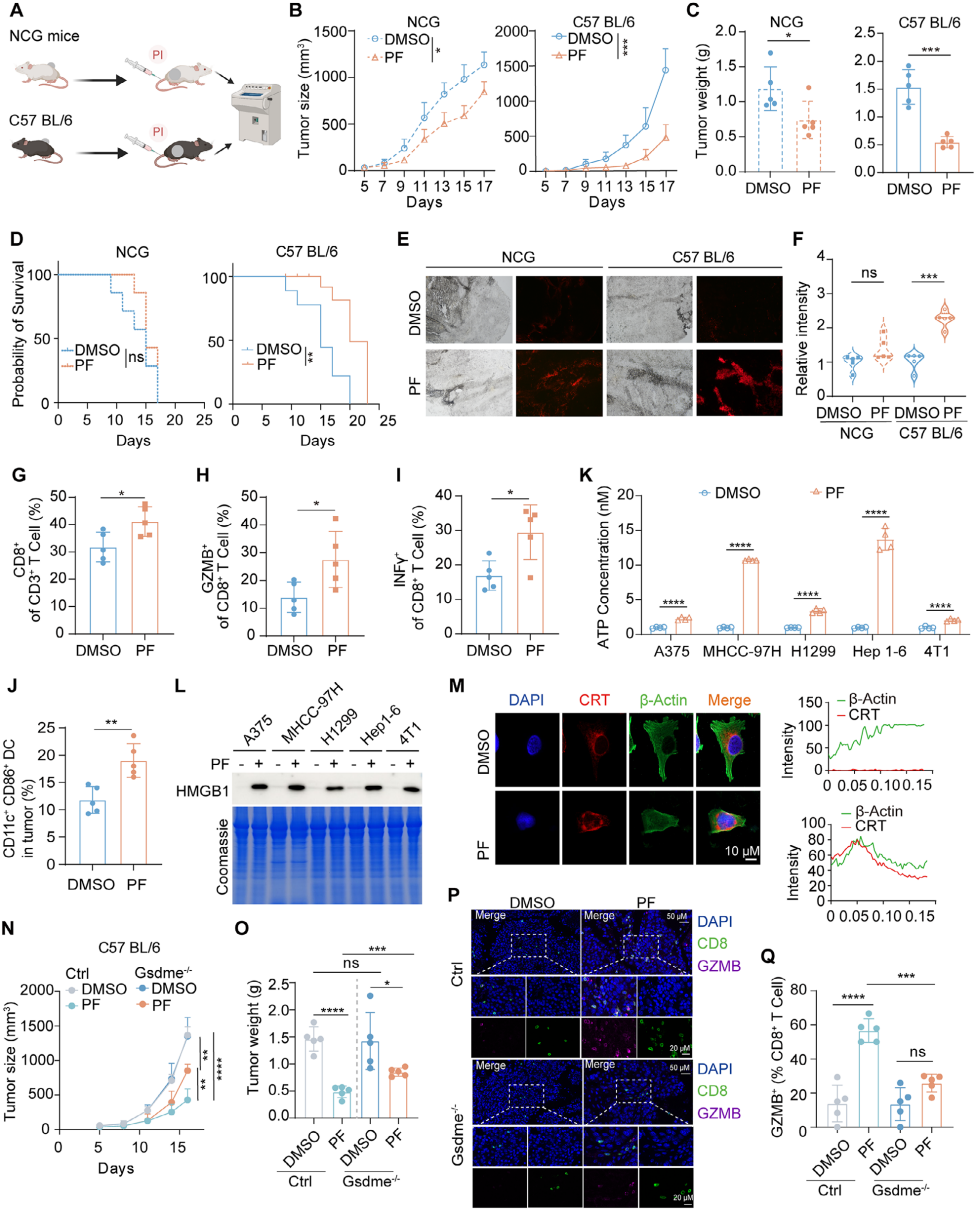
PF trigger immunogenic pyroptosis to activate anti‐tumor immunity. (A–D) Evaluation of B16F10 subcutaneous tumor growth in NCG and C57 BL/6 mice treated with DMSO or PF. (A) Experimental schematic; (B) Tumor growth curve; (C) Tumor weight; (D) Survival analysis. *n* = 5 mice per group. (E, F) Representative images of PI staining (E) and quantification analysis (F) of B16F10 subcutaneous tumors in NCG and C57 BL/6 mice after tumor inoculation. (G–I) The percentage of CD8^+^ T cells (G), CD8^+^ T cells expressing GZMB (H) and IFNγ (I) in B16F10 subcutaneous tumors from C57 BL/6 mice was analyzed by flow cytometry. (J) The percentage of intratumoral CD11c^+^ CD86^+^ dendritic cells in B16F10 subcutaneous tumors from C57 BL/6 mice was analyzed by flow cytometry. (K) Extracellular ATP levels were measured in indicated cells treated with 10 µm PF for 10 h. (L) HMGB1 levels in supernatants from indicated cells were detected, with Coomassie staining shown as a control. (M) Confocal microscopy images and line intensity plots showing CRT and β‐actin localization in HeLa cells to evaluate PF‐induced CRT translocation. (N, O) Tumor growth curve (N) and tumor weight (O) of B16F10 shCTRL and shGsdme subcutaneous tumor growth treated with DMSO or PF. (P, Q) Representative multiplex immunohistochemistry (mIHC) images of B16F10 tumor tissues (P) stained with the indicated antibody panel and quantitative analysis of the proportion of GZMB^+^ CD8^+^ cells (Q) in C57 BL/6 mice treated with PF.

Pyroptosis is reported to be an immunogenic and inflammatory cell death [35]. Given that PF selectively induces pyroptosis in tumor cells in vitro, we compared PF treatment in immunocompetent WT mice versus immunodeficient NCG mice (which lack functional lymphocytes) in order to differentiate between PF's direct cytotoxicity and the immune‑dependent component of tumor control (Figure 4A). As expected, PF moderately inhibited tumor growth and burden in NCG mice, but unexpectedly resulted in marked tumor reduction in immunocompetent WT mice (Figure 4B,C). Consistently, PF markedly prolonged survival in immunocompetent WT mice compared with immunodeficient NCG mice (Figure 4D), without causing body weight loss (Figure S4A,B). To compare pyroptotic activity between immunocompetent and immunodeficient mice, we performed in vivo PI uptake assays in NCG and C57 BL/6 mice. Notably, PF induced significantly greater cell death in tumors from WT mice than in those from NCG mice, indicating that the immune system contributes to PF‐induced cell death (Figure 4E,F). We hypothesize that PF initiates a feedforward loop wherein pyroptosis recruits and activates immune cells, which in turn promote further tumor killing. This hypothesis was supported by the observation that tumor‐infiltrating CD8^+^ T cells and their effect subtypes, indicated by IFNγ and GZMB expression level, were increased in response to PF treatment (Figure 4G–I; Figure S4C–E). Notably, PF treatment did not significantly alter the infiltration of CD4^+^ T cells, NK1.1^+^ cells, or regulatory T cells (Tregs) (Figure S4F–I).

The induction of ICD in cancer cells promotes their uptake by dendritic cells, which in turn elicits tumor antigen presentation and stimulates tumor‑specific cytotoxic T lymphocyte responses [36]. We therefore detected the abundance of dendritic cells by flow cytometry, finding that PF treatment increased the maturation of dendritic cells, indicated by elevated co‐expression of CD80 and CD86 on CD11c^+^ cells (Figure 4J; Figure S4J). Furthermore, we revealed that the ICD score was elevated and a series of ICD‐related pathways, including interferon signaling, antigen presentation, and endoplasmic reticulum stress pathways, were activated after PF treatment (Figure S3K,L). To biochemically validate ICD induction, we quantified the following canonical DAMPs: surface‐exposed calreticulin (CRT), HMGB1 mobilization, and ATP secretion. In contrast to the control group, cancer cells treated with PF had the higher level of ATP secretion (Figure 4K). Meanwhile, almost all HMGB1 were translocated to the extracellular environment (Figure 4L), and CRT translocated to the plasma membrane (Figure 4M). To directly test whether Gsdme‑mediated pyroptosis drives PF‑induced immune activation, we treated shGsdme tumors with PF in immunocompetent mice. Gsdme knockdown significantly reduced PF's antitumor effect (Figure 4N,O; Figure S4M,N) and largely abolished the increase in GZMB^+^ CD8^+^ T cells (Figure 4P,Q). Given that Gsdme is the executioner of pyroptosis, these findings provide direct causal evidence that PF exerts its antitumor immune effects primarily through Gsdme‑dependent pyroptosis. Thus, PF‑induced Gsdme‑mediated pyroptosis is dominant for immune activation and therapeutic efficacy in vivo.

### 2.5 PF Induces Tumor Regression and Enhances Antitumor Immunity in Spontaneous Mouse Models and Hydrodynamic Tail Vein Injection (HTVi)‐Induced HCC

**FIGURE 5:**
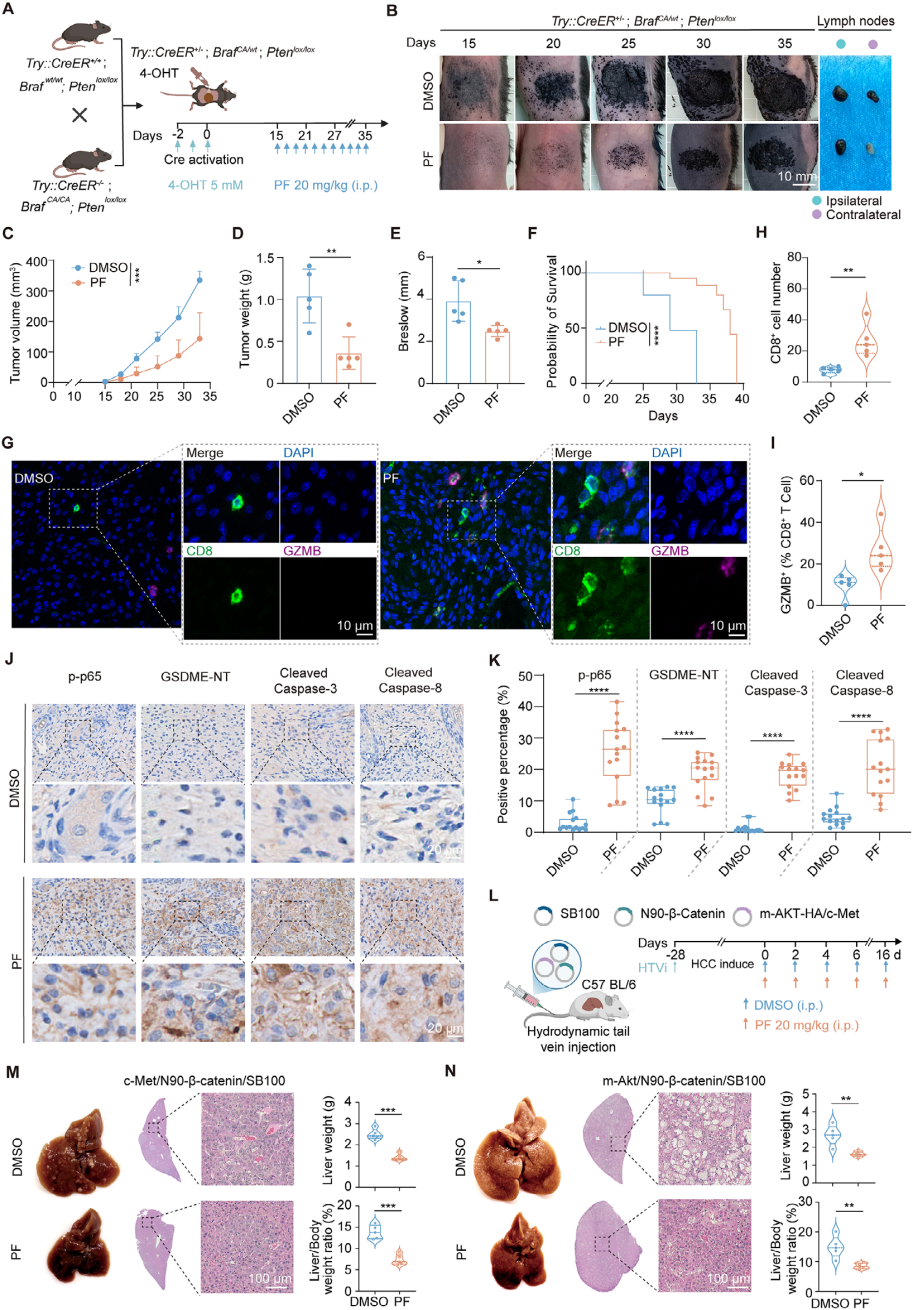
PF induces tumor regression and enhances antitumor immunity in spontaneous mouse models and hydrodynamic tail vein injection (HTVi)‐induced HCC. (A) Schematic administration of 20 mg/kg PF in Braf/Pten‐driven spontaneous melanoma mice. *n* = 5 per group. (B) Representative images of tumor regions and lymph nodes of Braf/Pten‐driven spontaneous melanoma mice at the indicated days post‐treatment. (C–F) Evaluation of Braf/Pten‐driven spontaneous melanoma in mice treated with DMSO or PF; (C) Tumor growth curve; (D) Tumor weight; (E) Breslow thickness analysis; (F) Survival curves; *n* = 5 mice per group. (G) Representative mIHC images of tumor tissues stained with the indicated antibody panel. (H, I) Quantification of frequency of CD8^+^ T cells (H) and GZMB^+^ proportions of tumor‐infiltrating CD8^+^ T cells (I) in Braf/Pten‐driven spontaneous melanoma mice treated with DMSO or PF. *n* = 5 mice per group. (J, K) Representative images (J) and quantification analysis (K) of p‐p65, GSDME‐NT, Cleaved Caspase‐3, Cleaved Caspase‐8 immunohistochemical staining Braf/Pten‐driven spontaneous melanoma mice treated with DMSO or PF. *n* = 5 mice per group. (L) Schematic administration of 20 mg/kg PF in hydrodynamic tail vein injection (HTVi)‐induced HCC. (M, N) Representative images of liver tumor tissues and HE staining, liver weight, and liver‐to‐body ratio of each group in the c‐Met/N90‐β‐catenin/SB100 (M) and m‐Akt/N90‐β‐catenin/SB100 (N) induced HDTVi model.

To further evaluate the therapeutic potential of PF treatment, we used a genetically engineered mouse model (Tyr::CreER^+/−^; Braf*^CA/WT^*, Pten*^lox/lox^*) that recapitulates spontaneous melanoma development upon activation of BrafV600E expression and Pten deletion (Figure 5A) [37]. PF treatment significantly reduced tumor growth and burden, and resulted in less pigmented lymph nodes at the endpoint (Figure 5B–D), without causing weight loss or apparent organ toxicity (Figure S5A,B). Breslow thickness, a key prognostic factor in melanoma, was significantly reduced by PF (Figure 5E). Importantly, PF enhanced overall survival in this murine melanoma model driven by Braf/Pten (Figure 5F). Quantitative analysis of mIHC indicated increased density of GZMB^+^ cytotoxic CD8^+^ T cells in treated tumors (Figure 5G–I). Moreover, we also observed that PF treatment resulted in the increased NFKB transcriptional activity, evidenced by increased levels of phospho‐p65 (p‐p65) expression, alongside a notable rise in cleavage of caspase‐8/‐3 and GSDME (Figure 5J,K), consistent with our in vitro mechanistic data. These results demonstrate that PF induces tumor regression and enhances anti‐tumor immunity through pyroptosis induction in a spontaneous melanoma model.

To further validate the therapeutic potential of PF in hepatocellular carcinoma (HCC), we utilized a hydrodynamic tail‐vein injection (HDVi)‐induced de novo hepatocarcinogenesis model (Figure 5L). PF treatment significantly inhibited HCC development, as evidenced by a marked reduction in both tumor nodule count and liver weight. Consistent with this, the liver‐to‐body weight ratio further indicated that the efficacy of PF was critical in restraining tumor progression (Figure 5M, N). Collectively, these results demonstrate that PF potently suppresses HCC initiation and growth.

#### 2.5.1 PF‐Induced Pyroptosis Potentiates Anti‐PD‐1 Immunotherapy

**FIGURE 6:**
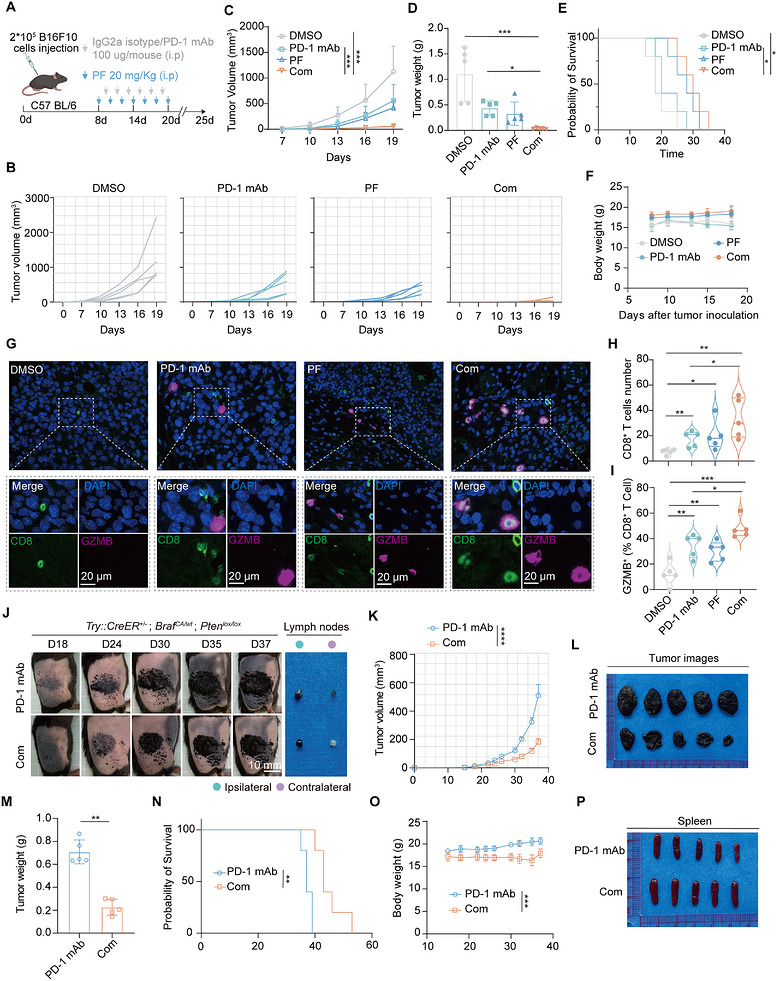
PF‐induced pyroptosis potentiates anti‐PD‐1 immunotherapy. (A–F) Analysis of B16F10 subcutaneous tumor model in C57 BL/6 mice following treatment with PF, PD‐1 mAb, or their combination; (A) Schematic illustration; (B, C) Tumor growth curves; (D) Tumor weight; (E) Survival curves; (F) Body weight. n = 5 mice per group. (G) Representative mIHC images of B16F10 tumor tissues stained with the indicated antibody panel. (H, I) Quantitative analysis of tumor‐infiltrating CD8^+^ T cells (H), and the proportion of GZMB^+^ CD8^+^ cells (I) in C57 BL/6 mice treated with PF, PD‐1 mAb, or their combination. n = 5 mice per group. (J) Representative images of tumor regions and lymph nodes from Braf/Pten‐driven spontaneous melanoma mice at the indicated days after treatment with PD‐1 mAb alone or in combination with PF. (K–P) Analysis of Braf/Pten‐driven spontaneous melanoma model in C57 BL/6 mice treated with PD‐1 mAb alone or in combination with PF. (K) Tumor growth curve; (L) Representative tumor images; (M) Tumor weight; (N) Survival curves; (O) Body weight; (P) Spleen volume *n* = 5 mice per group.3.

We next asked whether PF could improve response to anti‐programmed cell death 1 (anti‐PD‐1) immunotherapy. For this purpose, we evaluated a combinatorial approach that pairs PF with anti‑PD‑1 immunotherapy in the B16F10 mouse model. C57 BL/6 mice with palpable orthotopic tumors received vehicle control, anti‑PD‑1 alone, PF alone, or the combination of both (Figure 6A). As expected, moderate suppression of tumor growth was observed with anti‑PD‑1 monotherapy. In contrast, the combination of anti‑PD‑1 with PF produced a marked decrease in tumor growth and burden relative to anti‑PD‑1 or PF alone (Figure 6B–D). Importantly, combination therapy significantly prolonged survival without affecting body weight (Figure 6E,F). We further applied mIHC to characterize the modulatory effect of PF on the anti‑PD‑1‑elicited antitumor immune response. Anti‑PD‑1 monotherapy, as shown by the analysis, enhanced the effector function of tumor‑infiltrating CD8^+^ T cells, evidenced by increased GZMB expression. Notably, this effect was significantly potentiated when anti‑PD‑1 was combined with PF (Figure 6G–I).

We further validated these findings in the spontaneous Braf/Pten‐driven melanomas model, recapitulating an authentic tumor microenvironment. Consistent with previous observations, the combination of PF with anti‐PD‐1 elicited superior tumor suppression and significantly prolonged overall survival compared to anti‐PD‐1 monotherapy, again without body weight loss (Figure 6J–O). Mice receiving combination therapy developed splenomegaly (Figure 6P), indicative of systemic immune activation. Taken together, these findings demonstrate that PF significantly strengthens anti‑PD‑1 efficacy in subcutaneous and spontaneous melanoma mouse models.

### 2.6 PF Targets Correlated with the Inflammatory Responses and the Efficacy of Improved Immunotherapy

**FIGURE 7:**
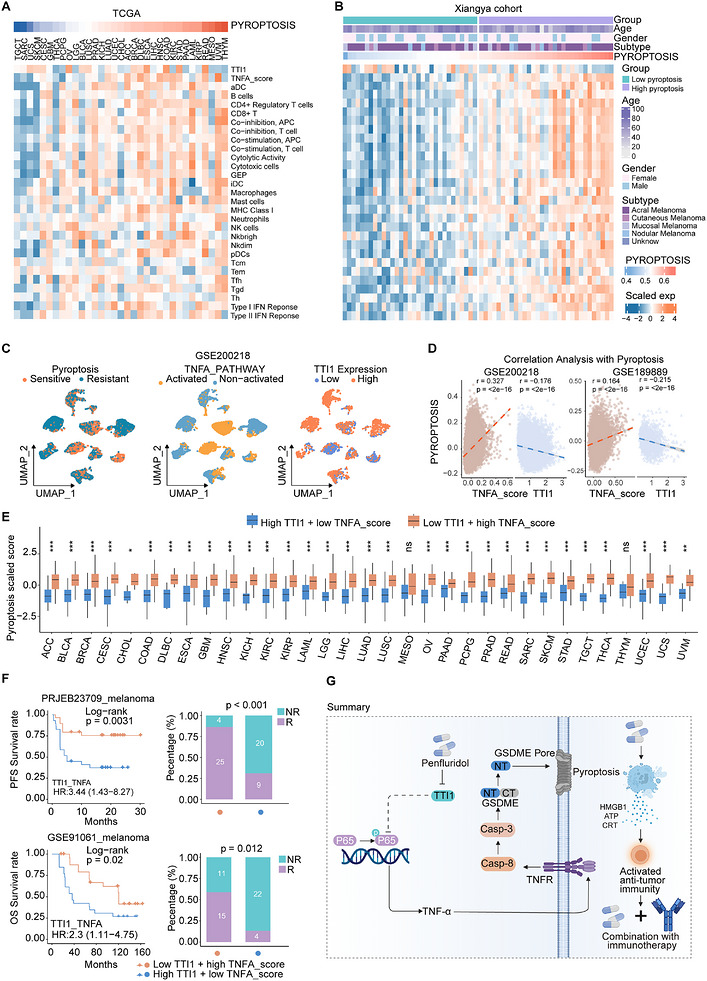
PF targets correlated with the inflammatory responses and the efficacy of improved immunotherapy. (A) Tumor‐specific differential analysis of immune infiltration signatures and pyroptosis in cancer patients stratified by TTI1 expression/ TNFA_score (High TTI1 + Low TNFA_score vs. Low TTI1 + High TNFA_score). (B) Heatmap showing the normalized pyroptosis score and immune infiltration pathway enrichment scores of each sample calculated by single‐sample GSEA (xiangya cohort). (C) t‐SNE analysis of melanoma scRNA‐seq data depicts tumor cell clustering, stratified by pyroptosis sensitivity, TNFA pathway activation status, and TTI1 expression levels in GSE200218 dataset. (D) Scatter plots show the Spearman correlation between the pyroptosis score and TNFA_score or TTI1 expression. (E) Pyroptosis activity scores in TCGA tumor patients stratified by TTI1 expression/ TNFA_score (High TTI1 + Low TNFA_score vs. Low TTI1 + High TNFA_score groups). (F) Kaplan Meier curves compare overall survival between High TTI1 + Low TNFA_score and Low TTI1 + High TNFA_score groups (left), and the proportion of patients with different responses to immunotherapy (right) in two independent ICI cohorts (PRJEB23709, GSE91061). (G) Schematic summary for the findings in the present study.

To further elucidate the role of PF targets in clinical samples, we analyzed the TCGA database and observed that patients with higher pyroptosis scores displayed significantly reduced TTI1 expression, elevated TNFA scores, and enhanced immune cell infiltration across multiple cancer types (Figure 7A). These findings were corroborated in an internal Xiangya Hospital cohort as well as an independent cohort of 437 patients and Ribas’ cohort (Figure 7B; Figure S6A–C). To dissect the association between pyroptosis activity and TTI1 expression or TNFA scores specifically within tumor cells, we turned to two independent melanoma single‐cell datasets (Figure S7A,B). After identifying malignant cells, we classified them into pyroptosis‐sensitive and pyroptosis‐resistant subpopulations. Notably, the pyroptosis‐sensitive subset largely overlapped with populations exhibiting TNFA pathway activation and low TTI1 expression (Figure 7C; Figure S7C). Furthermore, pyroptosis scores were positively correlated with TNFA pathway activity and inversely correlated with TTI1 expression (Figure 7D).

Based on these observations, we constructed a novel composite index integrating TNFA scores and TTI1 expression. In TCGA cohorts, patients with low TTI1 expression and high TNFA scores consistently showed elevated pyroptosis levels (Figure 7E). When applied to two independent immunotherapy datasets, this composite index revealed that patients characterized by low TTI1 and high TNFA experienced significantly prolonged overall survival and improved therapeutic response (Figure 7F).

## 3 Discussion

GSDM‐mediated pyroptosis has emerged as a key mechanism that elicits protective immunity and enhances the efficacy of immunotherapy [5]. While the role of pyroptosis in antitumor immunity is increasingly recognized, whether FDA‐approved antipsychotic drugs can trigger this form of ICD remained unexplored. In this study, we identify the antipsychotic agent PF as a potent inducer of pyroptosis via a previously unreported molecular pathway. Mechanistically, PF directly binds to and inhibits TTI1, leading to activation of the TNFA signaling via NFKB and subsequent caspase‐8/caspase‐3‐dependent cleavage of GSDME, culminating in pyroptotic cell death. Preclinically, PF not only exhibits monotherapy efficacy in immunocompetent hosts but also acts synergistically with anti‐PD‐1 therapy in both transplanted and spontaneous melanoma and HCC models without inducing systemic toxicity. Clinically, low TTI1 expression coupled with activated TNFA signaling via NFKB correlates with improved immunotherapy response and prolonged overall survival, suggesting its potential utility as a predictive biomarker (Figure 7G). Collectively, our work establishes a compelling paradigm for repurposing pyroptosis inducers to stimulate antitumor immunity.

PF, a first‐generation diphenylbutylpiperidine antipsychotic, has previously demonstrated anti‐cancer activity through direct tumor suppression [22], including suppression of triple‐negative breast cancer growth and brain metastasis via inhibition of integrin signaling and induction of apoptosis [29]; induction of apoptosis and autophagy in acute myeloid leukemia through PP2A‐mediated suppression of Akt/MAPK pathways and ROS accumulation [31]; targeting of PFKL to suppress glycolysis and tumorigenesis in esophageal cancer [30]; inhibition of melanoma progression via enhanced VHL‐CIP2A interaction [32]; and disruption of the KEAP1‐NRF2 axis to suppress MYC‐driven liver cancer [38]. Beyond its direct tumor‐suppressive effects, PF has been reported to modulate antitumor immunity through several distinct mechanisms, including reduction of myeloid‐derived suppressor cells (MDSCs)—established drivers of glioblastoma progression—with concurrent inhibition of Treg infiltration, enhancement of M1 macrophage polarization, and suppression of IFNγ/CCL4 [39]; remodeling of PD‐L1 glycosylation to disrupt PD‐1/PD‐L1 interactions, thereby activating T‐cell immunity [40]; and AMPK activation‐mediated proteasomal degradation of PD‐L1, driving its downregulation and promoting CD4^+^/CD8^+^ T‐cell infiltration [41]. Despite these insights, whether PF induces pyroptosis—an immunogenic form of cell death—to stimulate antitumor immunity remained unknown. Crucially, our study demonstrates for the first time that PF's antitumor effects are primarily mediated by pyroptosis‐induced ICD, expanding the mechanistic framework of how this antipsychotic drug exerts its antitumor effects.

Our findings position GSDME as the central executor of PF‐induced antitumor pyroptosis. Recent studies have substantially expanded the landscape of GSDME‐mediated pyroptosis in cancer. Endocytic pathways actively remove GSDME‐NT pores from the plasma membrane to restrain pyroptosis, exemplified by MYO6‐mediated endocytosis [42], MYO1G‐facilitated caveolin‐1‐dependent endocytosis [43], and TOLLIP‐targeted autophagic degradation of GSDME‐NT vesicles [44]. Conversely, direct pyroptosis induction kills tumor cells through alternative upstream signals, such as cGAS‐STING activation [45] or the PINK1‐inhibition‐ROS‐BAX‐GSDME axis [46]. Beyond direct cytotoxicity, triggering GSDME‐dependent pyroptosis has been shown to sensitize immunotherapy, as demonstrated by honokiol‐magnolol‐baicalin [47], platinum (IV) prodrugs [48], arachidonic acid [49], ascorbic acid [50], and the STAT1–GSDME circuitry [18]. Several aspects clarify the relationship between our findings and these studies. First, like the cGAS‐STING or PINK1‐ROS pathways, we induce GSDME‐dependent‐pyroptosis, but through a distinct upstream cascade—direct TTI1 inhibition leading to TNFA‐NFKB‐caspase‐8/3 activation—which has not been previously reported. Second, similar to other pyroptosis‐inducing agents that sensitize anti‐PD‐1 therapy, PF synergizes with immunotherapy specifically by triggering ICD. Thus, while sharing the downstream effector GSDME with these studies, our work uncovers a novel druggable target (TTI1) and a unique signaling axis. Finally, our clinical finding that low TTI1 expression correlates with improved immunotherapy response positions TTI1 as a potential predictive biomarker, complementing the growing understanding of pyroptosis‐based cancer therapy.

TTI1 has been implicated in oncogenesis, migration, and invasion of various cancers through regulation of the mTOR signaling pathway [51, 52, 53, 54]. We demonstrate a negative correlation between TTI1 expression and TNFA signaling via NFKB in cancer patient cohorts and establish that PF directly binds to and inhibits TTI1, leading to the activation of TNFA signaling via NFKB. However, the precise molecular mechanism by which TTI1 loss leads to NFKB activation remains unclear. Canonically, TTI1 is a component of the TTT complex that stabilizes PIKK family kinases, including ATM, ATR, and DNA‑PKcs, which are central to the DNA damage response (DDR) and cellular stress signaling [55, 56]. Emerging evidence suggests that DDR can extensively crosstalk with inflammatory pathways, particularly the NFKB axis, through mechanisms such as ATM‑mediated activation of IKK or ATR‑dependent paracrine signaling [57, 58, 59]. Thus, TTI1 loss could potentially destabilize these PIKKs and, via aberrant DDR activation, contribute to NFKB‑driven inflammatory responses. Further studies are warranted to directly test this possibility. This potential connection provides a broader context for understanding the TTI1‐NFKB axis identified in our study.

Regardless of the exact upstream mechanism, the activation of the TNFA‐NFKB axis—a central conduit of our proposed mechanism—has long been recognized as a “double‐edged sword” in tumor therapy [60]. On one hand, NFKB‐driven TNFA signaling can promote anti‐tumor immunity by inducing inflammatory cell death [61]. On the other hand, chronic or systemic activation of NFKB is frequently associated with tumor‐promoting inflammation, angiogenesis, and even resistance to apoptosis in cancer cells themselves [62, 63]. Moreover, RIPK1 can redirect TNF signaling to NFKB, which suppresses anti‐tumor immunity and reduces T/NK cell infiltration, ultimately leading to anti‐PD‐1 resistance [64]. This inherent complexity has historically hindered the clinical translation of strategies that broadly target this pathway. PF overcomes this paradox by directly binding and inhibiting TTI1, thereby triggering GSDME‑dependent pyroptosis specifically in cancer cells—unlike systemic TNFA activation or broad NFKB inhibition. This tumor‑restricted action harnesses the pathway's immunogenic potential while avoiding its systemic risks, achieving a therapeutic window previously elusive. The superiority of PF lies in leveraging the pathway's immunogenic potential while restricting its activity to the tumor, achieving a therapeutic window previously elusive.

While our findings reveal a novel mechanism by which PF induces pyroptosis and enhances anti‐tumor immunity, several limitations of this study warrant consideration. For example, our in vitro analyses were conducted in a limited set of cancer cell lines, and the in vivo validation was primarily performed in subcutaneous and spontaneous models of melanoma and liver cancer. These experimental systems may not fully recapitulate the complexity and heterogeneity of human tumor microenvironments. Future studies should seek to validate these findings in patient‐derived xenograft models of melanoma and across a broader spectrum of tumor types to assess generalizability. Furthermore, the clinical translation of PF must be carefully weighed against its known safety profile. As a potent antipsychotic, PF carries risks particularly relevant to cancer patients, including QT prolongation and associated arrhythmias, extrapyramidal symptoms, sedation, and hyperprolactinemia—a particular concern in hormone‐sensitive cancers [22, 23, 24]. Therefore, any future clinical evaluation of PF will require careful patient selection, proactive monitoring, and thorough risk‐benefit assessment.

Collectively, our findings uncover a new mechanism by which PF drives immunogenic pyroptosis, thus driving tumor‑targeting immune responses. These findings provide a strong preclinical foundation for repurposing this antipsychotic agent as a cost‐effective and safe combinational immunotherapeutic strategy, highlighting the potential of drug repurposing in expanding the arsenal of cancer immunotherapies.

## 4 Materials and Methods

### 4.1 Cell Culture

A panel of cell lines was used, including melanomas (A2058, WM35, A375, SK‐MEL‐5, SK‐MEL‐28, B16F10), Central nervous system cancer (U252, U87 MG, T98G), lung cancers (PC‐9, H1299), liver cancers (MHCC‐97H, LM3), breast cancers (MCF7, MDA‐MB‐231), genitourinary cancers (T24, 786‐O, UMUC3), gynecologic cancers (HeLa, SK‐OV‐3), PIG1, NHEK, and HEK293T cells, all purchased from the American Type Culture Collection. The human primary dermal fibroblasts were isolated from a normal skin sample obtained from a donor with written informed consent. All cell lines were cultured in their respective media: DMEM (Biological Industries) for most human lines and fibroblasts, and RPMI‐1640 (Biological Industries) for B16F10 cells. All media contained 10% fetal bovine serum (Biological Industries), with 1% penicillin–streptomycin (Beyotime Biotechnology) added. Cells were grown at 37 °C in a humid atmosphere containing 5% CO_2_.

### 4.2 Chemicals

PF (HY‐B1077), Ichloroquine (HY‐17589A), Ac‐DEVD‐CHO (HY‐P1001), necrostatin‐1s (HY‐14622A), Z‐VAD (HY‐16658B), Fer‐1 (HY‐100579), DL‐dithiothreitol (HY‐15917), Infliximab (HY‐P9970), Etanercept (HY‐108847) and Tetrathiomolybdate (HY‐128530) were purchased from MedChemExpress. All 240 screened antipsychotics compounds were purchased from Selleck (Table S1).

### 4.3 Cell Viability and Death Analysis

We measured cell viability with the CCK‐8 assay (Bimake, B34302), following a previously reported protocol [8]. To quantify cell death, cells were stained with propidium iodide (PI; Invitrogen, P1304MP) and examined either by fluorescence microscopy or flow cytometry.

For microscopic analysis, cells were seeded at 3 × 10^4^ per well in 24‑well plates. After the designated treatment, the cells were rinsed and exposed to 500 µL of PBS containing 5 µg/mL PI for 1 min, followed by immediate imaging on a fluorescence microscope (Nikon). For flow cytometric detection, cells were plated at 3 × 10^5^ per well in 6‑well plates. Post‑treatment, both floating and adherent cells were collected, resuspended in 200 µL of PI/PBS (5 µg/mL), and run on a BD flow cytometer.

### 4.4 3D Spheroids

To generate tumor spheroids, cells (10,000/well) were placed into 96‑well plates pre‑covered with 50 µL of 1.5% agarose (BioFroxx, Cat#1110GR100). After overnight aggregation, spheroids were treated as indicated, stained with 2 µg/mL PI for 30 min at 37 °C in the dark, and imaged using a fluorescence microscope.

### 4.5 LDH Measurement

A total of 6,000 cells were seeded per well in a 96‑well cell culture plate. After overnight incubation to allow cell adherence, the cells were treated with the indicated compounds. Upon completion of drug stimulation, the culture supernatant was collected directly for subsequent analysis. LDH activity was measured using the LDH Assay Kit from Beyotime Biotechnology (#C0019).

### 4.6 Colony Formation Assay

We seeded 5,000 cells per well in 6‑well plates, cells were cultured for 2–3 days before drug treatment. During the 14 days treatment period, the culture medium containing the specified concentrations of PF was refreshed every 3 days. Cells underwent fixation in 4% paraformaldehyde. They were then incubated with 0.5% crystal violet for 20 min and subsequently examined/analyzed.

### 4.7 Western Blot Analysis

We carried out western blotting as described earlier [8]. Proteins were visualized using an Odyssey Fc Imaging System (LI‐COR Biosciences). The following primary antibodies were used: GSDME (Abcam, ab215191), GSDMD (CST, 39754), Cleaved Caspase‐3 (CST, 9661T), Cleaved Caspase‐8 (CST, 9496T), Cleaved Caspase‐1(Adipogen, 43682304), Caspase‐3 (Proteinech, 19677‐1‐AP), Caspase‐8 (Proteinech, 13423‐1‐AP), Cleaved Caspase‐9 (CST, 7237T), PARP (CST, 9532S), Phospho‐NFKB p65 (Ser468) (Proteintech, 82335‐1‐RR), TTI1 (Proteintech, 22381‐1‐AP), Vinculin (Santa Cruz, sc‐73614), GAPDH (Proteintech, 60004‐1‐Ig), and β‐ACTIN (Proteintech, 66009‐1‐Ig).

### 4.8 RNA Extraction and Quantitative Real‐Time PCR

Total RNA was isolated with the SteadyPure Universal RNA Extraction Kit (AG21023) and reverse transcribed to cDNA using HiScript Q RT SuperMix (Vazyme, R223‐01), following the respective manufacturers’ protocols. qPCR was conducted on an Applied Biosystems QuantStudio 3 system (Thermo Fisher Scientific) with SYBR Green Master Mix (Bimake, B21703). Relative mRNA expression was calculated by the 2–ΔΔCt method, normalized to GAPDH, and primer sequences are provided in Table S2.

### 4.9 Plasmid Construction

Full‐length human GSDME (NM_001127454.2) and TTI1 (NM_001303457.1) coding sequences were amplified using Phanta Super‐fidelity DNA polymerase (Vazyme, #P501‐d1) and cloned into pLenti‐CMV‐MCS‐3FC‐Puro according to the ClonExpress II One Step Cloning Kit (Vazyme, Nanjing, China) and were identified by sequencing (TSINGKE, Beijing, China). The primers for amplification were GSDME: forward: ATAGAAGACACCGACTCTAGAGCCACCATGTTTGCCA

AAGC; Reverse: TTTGTAGTCAGCCCGGGATCCTGAATGTTCTCTGCCTAAAGCACA; TTI1: forward: ATAGAAGACACCGACTCTAGAATGGCAGTTTTTGATACTCCTGAGG; The sgRNA sequence targeting GSDME was 5′‐ACATGCCAGATGCTGCGCAT‐3′ and cloned into LentiCRISPRv2 vector. Reverse: TTTGTAGTCAGCCCGGGATCCCTGCAGCTCCTTGAGCAGCT; siCAS3: CCGAAAGGUGGCAACAGAAUUTT, siCAS8: GCCUUGAUGUUAUUCCAGAGATT, siCAS9: CCACUGCCUCAUUAUCAACAATT were provided by Banma Biotechnology Co., Ltd. (Changsha, China) and introduced into cells via Lipofectamine 3000 (Invitrogen, #3000075). The annealed oligos targeting the coding region were designed and cloned into pLKO.1 TRC cloning plasmid (Addgene). The sequence was: shCIP2A: TGCGGCACTTGGAGGTAATTT; shPFKL: GCTCCATCGATAACGAC

TTCT, shRELA: GATTGAGGAGAAACGTAAA, shTTI1: ATTGTCTATCTTCCCTAAA.

### 4.10 BLI

BLI experiments were performed on a GatorPrime instrument (GatorBio) at 30°C with shaking at 1000 rpm. Amine‐reactive APS biosensors (GatorBio) were used. Buffers: PBS, PBST (PBS + 0.05% Tween 20), and Q buffer (PBS + 0.02% Tween 20 + 0.2% BSA). APS sensors were baseline in PBS for 60 s, then loaded with 0.524 µg/mL PF (Selleck, S4151) in PBS for 200 s. After immobilization, the sensors were sequentially washed in PBS for 60 s, Q buffer for 120 s, and finally PBST for 120 s to remove nonspecifically bound molecules and achieve a stable baseline prior to association‐dissociation cycles. For kinetics, recombinant human TTI1 (Biodragon, BA07962) was twofold serially diluted in PBST (top concentration 1.25 µm). Association was performed for 120 s, followed by dissociation in PBST for 120 s. A nonspecific control (APS sensors without PF) was run under the same conditions. Data were analyzed using a 1:1 binding model to derive Kon and Koff, and Kd was calculated as Koff/Kon.

### 4.11 CETSA

Cells at ∼80% confluency in 10 cm dishes were treated with 10 µm PF or DMSO for 3 h at 37°C. Cells were harvested, resuspended in 800 µL PBS containing 1 mm PMSF, and aliquoted (100 µL) into seven tubes. Samples were heated from 43°C to 61°C in 3°C steps (3 min each), and equilibrated at RT. Then, to lyse cells, three cycles of freezing in liquid nitrogen and thawing were performed. After centrifugation at 12,000 rpm for 10 min, we collected the soluble supernatant, combined it with 5× SDS loading buffer, and then analyzed it by western blotting.

### 4.12 Subcutaneous Tumor Models

B16F10 melanoma cells (2 × 10^5^/mouse) were injected subcutaneously into the right flank of both immunodeficient NCG and immunocompetent C57BL/6 mice. Once tumors reached ∼100 mm^3^, mice were randomized to indicated treatments. Tumor dimensions were measured every three days with a digital caliper and calculated as (length × width^2^) × 0.5, with body weights recorded at each time point. The survival endpoint was defined as tumor volume exceeding 2,000 mm^3^.

### 4.13 Genetically Engineered Spontaneous Melanoma Models

For spontaneous melanoma induction, 3‑ to 5‑week‑old male Tyr::CreER^+^/^−^; Braf^CA^/^WT^; Pten^LOX^/^LOX^ mice received topical ethanol containing 2 µL of 5 mm 4‑OHT (Sigma‑Aldrich, #H6278) on shaved dorsal skin for three days (daily) to activate Cre. Tumor progression and monitoring were conducted according to previously established protocols [8].

### 4.14 HTVi‐Induced HCC

Six‐week‐old male C57 BL/6 mice (SJA Laboratory Animal Co. Ltd, Changsha) were used. A plasmid mixture containing pT3‑N90‐β‑CTNNB1 (Addgene #31785, 10 µg), pT3‐myr‐AKT‐HA (Addgene #31789, 20 µg), pT3‑EF1α‑Hc‑Met (Addgene #86498, 20 µg), and pCMV (CAT) T7‐SB100 (Addgene #34879) transposase plasmid (at 25% of the total plasmid mass) was diluted in 2 mL of 0.9% NaCl. The solution was administered through the tail vein over 5–8 s at a dose volume corresponding to 10% of the mouse body weight. Plasmid batch equivalency was confirmed to ensure reproducibility. Mice were monitored daily and humanely euthanized when exhibiting large abdominal tumors or signs of distress (e.g., lethargy, weight loss > 20%) according to institutional ethical guidelines. Solid tumors were harvested at endpoint and processed for hematoxylin and eosin (HE) staining analysis.

### 4.15 Multiplex Fluorescent Immunohistochemistry (mIHC)

mIHC was conducted in accordance with the manufacturer's instructions provided in the commercial assay kit (AiFang Biotechnology Co., Ltd.). Sequential staining was performed on tissue sections using primary antibodies targeting GZMB (Abcam, ab255598; diluted 1:1000) and CD8α (Abcam, ab217344; diluted 1:1000), with each antibody paired to an independent tyramide signal amplification system. Following each staining cycle, we performed antigen retrieval by incubating sections in sodium citrate buffer (pH 6.0; Servicebio, G1202) at high temperature for 20 min, which removed residual antibodies and eliminated cross‑reactivity. Nuclear staining was accomplished with DAPI (Abcam, ab104139).

### 4.16 Immunohistochemistry

Tissue sections were incubated overnight at 4°C with the following primary antibodies: anti‐GSDME (Proteintech, 13075‐1‐AP), anti‐Cleaved Caspase‐3 (CST, 9661T), anti‐Cleaved Caspase‐8 (Proteintech, 85171‐3‐RR), and anti‐Cleaved Phospho‐NFKB p65 (Ser468) (Proteintech, 82335‐1‐RR). We performed chromogenic detection with a DAB Substrate Kit (ZSGB‑Bio, ZLI‑9018) as instructed by the manufacturer. Light microscopy images of the sections were taken, and the staining intensity was measured using ImageJ.

### 4.17 In Vivo PI Staining Assay for Tumor Cell Death

Tumor‑bearing mice received drug treatment according to the designated protocol. One hour prior to euthanasia at the experimental endpoint, mice were intravenously administered PI at a dose of 2.5 mg/kg. Tumor tissues were then collected and embedded in OCT compound (Thermo Fisher Scientific) for cryopreservation. Tissue sections were cut at a thickness of 10 µm and observed under a fluorescence microscope (OLYMPUS, BX53).

### 4.18 RNA Sequencing and Transcriptome Analysis

Before RNA‑seq sample collection, melanoma cells were exposed to either DMSO or PF for 3 h or 6 h, with each experimental condition set up in triplicate. Following sequencing, raw reads were processed by adapter trimming, and quality control was conducted using FastQC (https://github.com/s‐andrews/FastQC). HISAT2 was employed for alignment of the clean reads against the GRCh38.p12 human reference genome. Transcript assembly and quantification were performed using StringTie in conjunction with the hg38_ucsc.annotated.gtf annotation file. Differential gene expression analysis was carried out with DESeq2, applying a significance threshold of |log_2_ fold change| ≥ log_2_ (1.5) and an adjusted p‐value < 0.05.

### 4.19 Data Collection

Transcriptomic and clinical data for diverse tumor samples were sourced from the Cancer Genome Atlas (TCGA) data portal. From the Gene Expression Omnibus (GEO; https://www.ncbi.nlm.nih.gov/geo/), datasets for an immunotherapy cohort (GSE91061) and melanoma single‐cell datasets (GSE20018 and GSE189889) were downloaded. We also utilized a transcriptomic dataset of 437 ICI‐treated advanced melanoma patients from Ribas et al. [65], and the Gide cohort [66] (PRJEB23709) from the Sequence Read Archive (SRA, https://www.ncbi.nlm.nih.gov/bioproject/), which contains data on anti‐PD‐1 mono‐ and combination therapy.

### 4.20 Quantification of Pathway Activity

The pyroptosis pathway activity score was calculated using the “ REACTOME_PYROPTOSIS ” gene set from the Molecular Signatures Database (MSigDB v7.5.1; https://www.gsea‐msigdb.org). Immune cell infiltration levels were estimated based on published gene signatures [67, 68, 69]. Using the GSVAR package (v1.44.2), single‐sample gene set enrichment analysis (ssGSEA) was applied to calculate per‐sample pathway enrichment scores.

### 4.21 Statistical Analysis

Pyroptosis scores across groups stratified by TTI1 expression or TNFA pathway activity were compared using the Wilcoxon rank‐sum test. Response rates between subgroups defined by TTI1 expression or TNFA pathway activity were assessed using Fisher's exact test or the χ^2^ test, as appropriate. Survival outcomes were assessed using the Kaplan‐Meier method, followed by statistical comparison with the log‐rank test.

### 4.22 Ethics Approval and Consent to Participate

The collection of clinical samples was approved by the Xiangya Hospital Institutional Review Board (No. 2022060825), and written informed consent was obtained from the donor. All animal studies were approved by the Xiangya Hospital Institutional Animal Care and Use Committee (No. 252512249) and conducted in accordance with institutional regulations.

## Author Contributions

Conceptualization: G. Deng, C. Zhang, and F. Zeng; Methodology: L. Yao, H. Su, Z. Guo, D. Zhao, and Z. Wu; Bioinformatics analysis: Y. Dian; Experiment: L. Li, D. Li, and D. Chen; Writing – original draft: L. Li, D. Li, and D. Chen; Writing – review & editing: G. Deng, C. Zhang, and F. Zeng.

## Funding

This work was supported by the National Natural Science Foundation of China (Grant Nos. 82301999 to L. Li, 82573574 to F. Zeng, and 82272849 to G. Deng), and Fundamental Research Funds for the Central Universities of Central South University (Grant No. 1053320240653 to D. Li).

## Conflicts of Interest

The authors declare no conflicts of interest.

## Supporting information




**Supporting File 1**: advs76408‐sup‐0001‐SuppMat.docx.

**Supporting File 2**: advs76408‐sup‐0002‐TableS1.xlsx.

## Data Availability

The data that support the findings of this study are available from the corresponding author upon reasonable request.

## References

[1] S. Jhunjhunwala , C. Hammer , and L. Delamarre , “Antigen Presentation in Cancer: Insights into Tumour Immunogenicity and Immune Evasion,” Nature reviews Cancer 21, no. 5 (2021): 298–312, 10.1038/s41568-021-00339-z. 33750922

[2] P. Fontana , G. Du , Y. Zhang , et al., “Small‐molecule GSDMD Agonism in Tumors Stimulates Antitumor Immunity without Toxicity,” Cell 187, no. 22 (2024): 6165–6181.e22, 10.1016/j.cell.2024.08.007. 39243763 PMC11648675

[3] C. Galassi , T. A. Chan , I. Vitale , and L. Galluzzi , “The Hallmarks of Cancer Immune Evasion,” Cancer Cell 42, no. 11 (2024): 1825–1863, 10.1016/j.ccell.2024.09.010. 39393356

[4] K. Peng , X. Zhao , Y. X. Fu , and Y. Liang , “Eliciting Antitumor Immunity via Therapeutic Cancer Vaccines,” Cellular & molecular immunology 22, no. 8 (2025): 840–868, 10.1038/s41423-025-01316-4. 40629076 PMC12311208

[5] Z. Guo , Y. Liu , D. Chen , et al., “Targeting Regulated Cell Death: Apoptosis, Necroptosis, Pyroptosis, Ferroptosis, and Cuproptosis in Anticancer Immunity,” Journal of translational internal medicine 13, no. 1 (2025): 10–32, 10.1515/jtim-2025-0004. 40115032 PMC11921819

[6] G. Kroemer , C. Galassi , L. Zitvogel , and L. Galluzzi , “Immunogenic Cell Stress and Death,” Nature immunology 23, no. 4 (2022): 487–500, 10.1038/s41590-022-01132-2. 35145297

[7] L. Galluzzi , E. Guilbaud , D. Schmidt , G. Kroemer , and F. M. Marincola , “Targeting Immunogenic Cell Stress and Death for Cancer Therapy,” Nature reviews Drug discovery 23, no. 6 (2024): 445–460, 10.1038/s41573-024-00920-9. 38622310 PMC11153000

[8] Q. Zhou , Y. Sun , S. Du , et al., “Vorapaxar Enhanced Mitochondria‐associated Ferroptosis Primes Cancer Immunotherapy via Targeting FOXO1/HMOX1 Axis,” Cell reports Medicine 6, no. 10 (2025): 102371, 10.1016/j.xcrm.2025.102371. 41005298 PMC12629800

[9] Q. Zhou , Y. Dian , Y. He , et al., “Propafenone Facilitates Mitochondrial‐associated Ferroptosis and Synergizes with Immunotherapy in Melanoma,” Journal for immunotherapy of cancer 12, no. 11 (2024): 009805, 10.1136/jitc-2024-009805. PMC11590812 39581704

[10] R. J. Sullivan and J. S. Weber , “Immune‐related Toxicities of Checkpoint Inhibitors: Mechanisms and Mitigation Strategies,” Nature reviews Drug discovery 21, no. 7 (2022): 495–508, 10.1038/s41573-021-00259-5. 34316029

[11] P. Broz , “Pyroptosis: Molecular Mechanisms and Roles in Disease,” Cell research 35, no. 5 (2025): 334–344, 10.1038/s41422-025-01107-6. 40181184 PMC12012027

[12] A. Alishvandi , C. Aram , F. F. Shahrivar , P. Kesharwani , and A. Sahebkar , “Pyroptosis in Cancer Therapy: A Double‐edged Sword for Immune Activation and Tumor Progression,” Molecular cancer 24, no. 1 (2025): 297, 10.1186/s12943-025-02506-4. 41291696 PMC12649103

[13] C. Huang , J. Li , R. Wu , Y. Li , and C. Zhang , “Targeting Pyroptosis for Cancer Immunotherapy: Mechanistic Insights and Clinical Perspectives,” Molecular cancer 24, no. 1 (2025): 131, 10.1186/s12943-025-02344-4. 40319304 PMC12049004

[14] Y. Wang , W. Gao , X. Shi , et al., “Chemotherapy Drugs Induce Pyroptosis through Caspase‐3 Cleavage of a Gasdermin,” Nature 547, no. 7661 (2017): 99–103, 10.1038/nature22393. 28459430

[15] M. Jiang , L. Qi , L. Li , and Y. Li , “The Caspase‐3/GSDME Signal Pathway as a Switch between Apoptosis and Pyroptosis in Cancer,” Cell death discovery 6 (2020): 112, 10.1038/s41420-020-00349-0. 33133646 PMC7595122

[16] F. Wu , M. Wang , T. Zhong , et al., “Inhibition of CDC20 Potentiates Anti‐tumor Immunity through Facilitating GSDME‐mediated Pyroptosis in Prostate Cancer,” Experimental hematology & oncology 12, no. 1 (2023): 67, 10.1186/s40164-023-00428-9. 37528490 PMC10391908

[17] Y. Ren , M. Feng , X. Hao , et al., “USP48 Stabilizes Gasdermin E to Promote Pyroptosis in Cancer,” Cancer research 83, no. 7 (2023): 1074–1093, 10.1158/0008-5472.CAN-22-1812. 36607699

[18] Y. Tu , H. Wu , C. Zhong , et al., “Pharmacological Activation of STAT1‐GSDME Pyroptotic Circuitry Reinforces Epigenetic Immunotherapy for Hepatocellular Carcinoma,” Gut 74, no. 4 (2025): 613–627, 10.1136/gutjnl-2024-332281. 39486886 PMC12013592

[19] S. Pushpakom , F. Iorio , P. A. Eyers , et al., “Drug Repurposing: Progress, Challenges and Recommendations,” Nature reviews Drug discovery 18, no. 1 (2019): 41–58, 10.1038/nrd.2018.168. 30310233

[20] M. I. De Silva , H. K. Gan , and C. Bardy , “Repurposing Trifluoperazine for Glioblastoma Treatment,” Trends in pharmacological sciences 46, no. 5 (2025): 392–406. 40300936 10.1016/j.tips.2025.03.005

[21] L. Fu , W. Jin , J. Zhang , et al., “Repurposing Non‐oncology Small‐molecule Drugs to Improve Cancer Therapy: Current Situation and Future Directions,” Acta pharmaceutica Sinica B 12, no. 2 (2022): 532–557, 10.1016/j.apsb.2021.09.006. 35256933 PMC8897051

[22] V. Shaw , S. Srivastava , and S. K. Srivastava , “Repurposing Antipsychotics of the Diphenylbutylpiperidine Class for Cancer Therapy,” Seminars in cancer biology 68 (2021): 75–83, 10.1016/j.semcancer.2019.10.007. 31618686 PMC7152558

[23] N. M. Tuan and C. H. Lee , “Penfluridol as a Candidate of Drug Repurposing for Anticancer Agent,” Molecules 24, no. 20 (2019): 3659, 10.3390/molecules24203659. 31614431 PMC6832311

[24] A. Ali Ibrahim Mze and A. Abdul Rahman , “Repurposing the Antipsychotic Drug Penfluridol for Cancer Treatment (Review),” Oncology reports 52, no. 6 (2024): 176. 10.3892/or.2024.8833 PMC11541647 39513619

[25] S. C. Smith , A. S. Baras , J. K. Lee , and D. Theodorescu , “The COXEN Principle: Translating Signatures of in Vitro Chemosensitivity into Tools for Clinical Outcome Prediction and Drug Discovery in Cancer,” Cancer research 70, no. 5 (2010): 1753–1758, 10.1158/0008-5472.CAN-09-3562. 20160033 PMC2831138

[26] Y. Bai , Y. Pan , and X. Liu , “Mechanistic Insights into Gasdermin‐mediated Pyroptosis,” Nature reviews Molecular cell biology 26, no. 7 (2025): 501–521, 10.1038/s41580-025-00837-0. 40128620

[27] J. Shi , Y. Zhao , K. Wang , et al., “Cleavage of GSDMD by Inflammatory Caspases Determines Pyroptotic Cell Death,” Nature 526, no. 7575 (2015): 660–665, 10.1038/nature15514. 26375003

[28] J. Pang and J. E. Vince , “The Role of Caspase‐8 in Inflammatory Signalling and Pyroptotic Cell Death,” Seminars in immunology 70 (2023): 101832, 10.1016/j.smim.2023.101832. 37625331

[29] A. Ranjan , P. Gupta , and S. K. Srivastava , “Penfluridol: An Antipsychotic Agent Suppresses Metastatic Tumor Growth in Triple‐Negative Breast Cancer by Inhibiting Integrin Signaling Axis,” Cancer research 76, no. 4 (2016): 877–890, 10.1158/0008-5472.CAN-15-1233. 26627008 PMC4755811

[30] C. Zheng , X. Yu , Y. Liang , et al., “Targeting PFKL with Penfluridol Inhibits Glycolysis and Suppresses Esophageal Cancer Tumorigenesis in an AMPK/FOXO3a/BIM‐dependent Manner,” Acta pharmaceutica Sinica B 12, no. 3 (2022): 1271–1287, 10.1016/j.apsb.2021.09.007. 35530161 PMC9069409

[31] S.‐Y. Wu , Y.‐C. Wen , C.‐C. Ku , et al., “Penfluridol Triggers Cytoprotective Autophagy and Cellular Apoptosis through ROS Induction and Activation of the PP2A‐modulated MAPK Pathway in Acute Myeloid Leukemia with Different FLT3 Statuses,” Journal of biomedical science 26, no. 1 (2019): 63, 10.1186/s12929-019-0557-2. 31470848 PMC6717358

[32] F. Xu , J. Li , M. Ai , et al., “Penfluridol Inhibits Melanoma Growth and Metastasis through Enhancing von Hippel‒Lindau Tumor Suppressor‐Mediated Cancerous Inhibitor of Protein Phosphatase 2A (CIP2A) Degradation,” MedComm 5, no. 10 (2024): 758, 10.1002/mco2.758. PMC11470999 39399646

[33] W.‐Y. Hung , J.‐H. Chang , Y. Cheng , et al., “Autophagosome Accumulation‐mediated ATP Energy Deprivation Induced by Penfluridol Triggers Nonapoptotic Cell Death of Lung Cancer via Activating Unfolded Protein Response,” Cell death & disease 10, no. 8 (2019): 538, 10.1038/s41419-019-1785-9. 31308361 PMC6629704

[34] Y. Xia , P. Huang , Y.‐Y. Qian , et al., “PARP Inhibitors Enhance Antitumor Immune Responses by Triggering Pyroptosis via TNF–caspase 8–GSDMD/E Axis in Ovarian Cancer,” Journal for immunotherapy of cancer 12, no. 10 (2024): 009032, 10.1136/jitc-2024-009032. PMC11459312 39366751

[35] R. Tang , J. Xu , B. Zhang , et al., “Ferroptosis, Necroptosis, and Pyroptosis in Anticancer Immunity,” Journal of hematology & oncology 13, no. 1 (2020): 110, 10.1186/s13045-020-00946-7. 32778143 PMC7418434

[36] N. Yatim , S. Cullen , and M. L. Albert , “Dying Cells Actively Regulate Adaptive Immune Responses,” Nature reviews Immunology 17, no. 4 (2017): 262–275, 10.1038/nri.2017.9. 28287107

[37] D. Dankort , D. P. Curley , R. A. Cartlidge , et al., “BrafV600E cooperates with Pten Loss to Induce Metastatic Melanoma,” Nature Genetics 41, no. 5 (2009): 544–552, 10.1038/ng.356. 19282848 PMC2705918

[38] M. T. Nguyen , G. J. Lee , B. Kim , et al., “Penfluridol Suppresses MYC‐driven ANLN Expression and Liver Cancer Progression by Disrupting the KEAP1–NRF2 Interaction,” Pharmacological Research 210 (2024): 107512, 10.1016/j.phrs.2024.107512. 39643070

[39] A. Ranjan , S. Wright , and S. K. Srivastava , “Immune Consequences of Penfluridol Treatment Associated with Inhibition of Glioblastoma Tumor Growth,” Oncotarget 8, no. 29 (2017): 47632–47641, 10.18632/oncotarget.17425. 28512255 PMC5564593

[40] W. Xu , Y. Wang , N. Zhang , et al., “The Antipsychotic Drug Penfluridol Inhibits N‐Linked Glycoprotein Processing and Enhances T‐cell–Mediated Tumor Immunity,” Molecular Cancer Therapeutics 23, no. 5 (2024): 648–661, 10.1158/1535-7163.MCT-23-0449. 37963566

[41] J. Wang , Y. Zhang , Q. Chen , X. Wang , R. Cui , and P. Hou , “Penfluridol Enhances Anti‐Tumor Immunity in Colorectal Cancer by Inducing Proteasome‐mediated Degradation of PD‐L1 via the Activation of AMPK,” Translational Oncology 62 (2025): 102559, 10.1016/j.tranon.2025.102559. 41045641 PMC12529539

[42] J. Wu , C. Ding , C. Zhang , et al., “Methionine Metabolite Spermidine Inhibits Tumor Pyroptosis by Enhancing MYO6‐mediated Endocytosis,” Nature Communications 16, no. 1 (2025): 2184, 10.1038/s41467-025-57511-4. PMC11880502 40038267

[43] C. Ding , J. Wu , Z. Xu , et al., “MYO1G Facilitates Caveolin 1–Mediated Endocytosis of the N‐Terminal Fragments of Gasdermin E to Restrain Pyroptosis and Chemotherapy Efficacy,” Cancer Research (2026): OF1–OF17, 10.1158/0008-5472.CAN-25-0786. 42133406

[44] Z. Xu , Z. Li , Z. Deng , et al., “TOLLIP Targets GSDME‐NT‐carrying Endocytic Vesicles for Autophagy to Regulate Pyroptosis and Chemotherapy Efficacy,” Nature Cell Biology 28, no. 4 (2026): 812–827, 10.1038/s41556-026-01902-2. 41803502

[45] L. Xiao , Y.‐L. Ai , X.‐Y. Mi , et al., “cGAS Activation Converges with Intracellular Acidification to Promote STING Aggregation and Pyroptosis in Tumor Models,” Journal of Clinical Investigation 135, no. 18 (2025): 188872, 10.1172/JCI188872. PMC12435844 40663398

[46] Y. Zhu , M. Cao , Y. Tang , et al., “Inhibition of PINK1 Senses ROS Signaling to Facilitate Neuroblastoma Cell Pyroptosis,” Autophagy 21, no. 10 (2025): 2091–2110, 10.1080/15548627.2025.2487037. 40160153 PMC12459364

[47] Q. Gao , Q. Sheng , Z. Yang , et al., “Honokiol‐Magnolol‐Baicalin Possesses Synergistic Anticancer Potential and Enhances the Efficacy of Anti‐PD‐1 Immunotherapy in Colorectal Cancer by Triggering GSDME‐Dependent Pyroptosis,” Advanced Science 12, no. 13 (2025): 2417022, 10.1002/advs.202417022. 39950759 PMC11967828

[48] X. Yang , C. Xu , Y. Zeng , et al., “Pyroptosis‐Inducing Platinum(IV) Prodrugs via GSDME Pathway for Chemoimmunotherapy and Metastasis Inhibition in Triple‐Negative Breast Cancer,” Advanced Science 12, no. 29 (2025): 05567, 10.1002/advs.202505567. PMC12362741 40432601

[49] T. Chu , Z. Liu , H. Liu , et al., “Arachidonic Acid Induces Pyroptosis via a Non‐autophagic Function of Mitophagy and Enhances Immunotherapy in a PDAC Model,” Nature Communications 17, no. 1 (2026): 1545, 10.1038/s41467-025-68267-2. PMC12894901 41519804

[50] X. Sun , X. Cai , S. Li , et al., “High‐dose Ascorbic Acid Selectively Induces Pyroptosis in LKB1‐deficient Lung Cancer and Sensitizes Immunotherapy,” Cell Reports Medicine 6, no. 9 (2025): 102291, 10.1016/j.xcrm.2025.102291. 40818456 PMC12490232

[51] V. Fernández‐Sáiz , B.‐S. Targosz , S. Lemeer , et al., “SCFFbxo9 and CK2 Direct the Cellular Response to Growth Factor Withdrawal via Tel2/Tti1 Degradation and Promote Survival in Multiple Myeloma,” Nature Cell Biology 15, no. 1 (2013): 72–81, 10.1038/ncb2651. 23263282

[52] L.‐X. Zhang , X. Yang , Z.‐B. Wu , et al., “TTI1 promotes Non‐Small‐Cell Lung Cancer Progression by Regulating the mTOR Signaling Pathway,” Cancer Science 114, no. 3 (2023): 855–869, 10.1111/cas.15668. 36403197 PMC9986064

[53] P. Xu , G. Du , H. Guan , W. Xiao , L. Sun , and H. Yang , “A Role of TTI1 in the Colorectal Cancer by Promoting Proliferation,” Translational Cancer Research 10, no. 3 (2021): 1378–1388, 10.21037/tcr-20-3322. 35116463 PMC8798799

[54] J. Wang , L. Li , Y. Qin , and W. Zhang , “TTI1 contributes to Radioresistance by Activating ATM Pathway in Rectal Cancer,” Journal of Translational Medicine 23, no. 1 (2025): 652, 10.1186/s12967-025-06648-3. 40514657 PMC12166592

[55] M. Pal , H. Muñoz‐Hernandez , D. Bjorklund , et al., “Structure of the TELO2‐TTI1‐TTI2 Complex and Its Function in TOR Recruitment to the R2TP Chaperone,” Cell reports 36, no. 1 (2021): 109317, 10.1016/j.celrep.2021.109317. 34233195 PMC8278493

[56] K. E. Hurov , C. Cotta‐Ramusino , and S. J. Elledge , “A Genetic Screen Identifies the Triple T Complex Required for DNA Damage Signaling and ATM and ATR Stability,” Genes & Development 24, no. 17 (2010): 1939–1950, 10.1101/gad.1934210. 20810650 PMC2932975

[57] E. Bournique , A. Sanchez , S. Oh , et al., “ATM and IRAK1 Orchestrate Two Distinct Mechanisms of NF‐κB Activation in Response to DNA Damage,” Nature Structural & Molecular Biology 32, no. 4 (2025): 740–755, 10.1038/s41594-024-01417-0. PMC11997730 39753776

[58] M. Hinz , M. Stilmann , S. Ç. Arslan , K. K. Khanna , G. Dittmar , and C. Scheidereit , “A Cytoplasmic ATM‐TRAF6‐cIAP1 Module Links Nuclear DNA Damage Signaling to Ubiquitin‐Mediated NF‐κB Activation,” Molecular Cell 40, no. 1 (2010): 63–74, 10.1016/j.molcel.2010.09.008. 20932475

[59] Z. H. Wu and S. Miyamoto , “Induction of a Pro‐Apoptotic ATM–NF‐κB Pathway and Its Repression by ATR in Response to Replication Stress,” The EMBO Journal 27, no. 14 (2008): 1963–1973, 10.1038/emboj.2008.127. 18583959 PMC2486281

[60] Q. Guo , Y. Jin , X. Chen , et al., “NF‐κB in Biology and Targeted Therapy: New Insights and Translational Implications,” Signal Transduction and Targeted Therapy 9, no. 1 (2024): 53, 10.1038/s41392-024-01757-9. 38433280 PMC10910037

[61] W. Gao , X. Wang , Y. Zhou , X. Wang , and Y. Yu , “Autophagy, Ferroptosis, Pyroptosis, and Necroptosis in Tumor Immunotherapy,” Signal Transduction and Targeted Therapy 7, no. 1 (2022): 196, 10.1038/s41392-022-01046-3. 35725836 PMC9208265

[62] S. Ahmad , M. Abbas , M. F. Ullah , et al., “Long Non‐coding RNAs Regulated NF‐κB Signaling in Cancer Metastasis: Micromanaging by Not so Small Non‐coding RNAs,” Seminars in Cancer Biology 85 (2022): 155–163, 10.1016/j.semcancer.2021.07.015. 34314819

[63] E. Elinav , R. Nowarski , C. A. Thaiss , B. Hu , C. Jin , and R. A. Flavell , “Inflammation‐induced Cancer: Crosstalk between Tumours, Immune Cells and Microorganisms,” Nature Reviews Cancer 13, no. 11 (2013): 759–771, 10.1038/nrc3611. 24154716

[64] L. Cucolo , Q. Chen , J. Qiu , et al., “The Interferon‐stimulated Gene RIPK1 Regulates Cancer Cell Intrinsic and Extrinsic Resistance to Immune Checkpoint Blockade,” Immunity 55, no. 4 (2022): 671–685.e10, 10.1016/j.immuni.2022.03.007. 35417675 PMC11289737

[65] K. M. Campbell , M. Amouzgar , S. M. Pfeiffer , et al., “Prior Anti‐CTLA‐4 Therapy Impacts Molecular Characteristics Associated with anti‐PD‐1 Response in Advanced Melanoma,” Cancer Cell 41, no. 4 (2023): 791–806.e4, 10.1016/j.ccell.2023.03.010. 37037616 PMC10187051

[66] T. N. Gide , C. Quek , A. M. Menzies , et al., “Distinct Immune Cell Populations Define Response to Anti‐PD‐1 Monotherapy and Anti‐PD‐1/Anti‐CTLA‐4 Combined Therapy,” Cancer Cell 35, no. 2 (2019): 238–255.e6, 10.1016/j.ccell.2019.01.003. 30753825

[67] D. Tamborero , C. Rubio‐Perez , F. Muiños , et al., “A Pan‐cancer Landscape of Interactions Between Solid Tumors and Infiltrating Immune Cell Populations,” Clinical Cancer Research: An Official Journal of the American Association for Cancer Research 24 (2018), 3717–3728. 29666300 10.1158/1078-0432.CCR-17-3509

[68] M. Ayers , J. Lunceford , M. Nebozhyn , et al., “IFN‐γ–related mRNA Profile Predicts Clinical Response to PD‐1 Blockade,” Journal of Clinical Investigation 127, no. 8 (2017): 2930–2940, 10.1172/JCI91190. 28650338 PMC5531419

[69] M. S. Rooney , S. A. Shukla , C. J. Wu , G. Getz , and N. Hacohen , “Molecular and Genetic Properties of Tumors Associated with Local Immune Cytolytic Activity,” Cell 160, no. 1‐2 (2015): 48–61, 10.1016/j.cell.2014.12.033. 25594174 PMC4856474

