# Supplementary material for: Penfluridol Triggers GSDME‐Mediated Immunogenic Pyroptosis to Potentiate Antitumor Immunotherapy
Source: Adv Sci (Weinh). 2026 Jul 3;13(54):e76408. doi: 10.1002/advs.76408 (PMC13334586; doi:10.1002/advs.76408)
Supplement: Supplementary file 1 [file ADVS-13-e76408-s002.docx]

Supplemental information

**Penfluridol Triggers GSDME-Mediated Immunogenic Pyroptosis to potentiate Antitumor Immunotherapy**

*Linfeng Li, Daishi Li, Danyao Chen, Yating Dian, Lei Yao, Hui Su, Ziyu Guo, Deze Zhao, Zihua Wu, Furong Zeng^*^,* *Chunfang* *Zhang^*^,* *and Guangtong Deng^*^*

**Table S2.** **Sequence of primers used for real-time PCR.**

| Gene symbol | Forward primer | Reverse primer |
| --- | --- | --- |
| Caspase-3 | GAAATTGTGGAATTGATGCGTGA | CTACAACGATCCCCTCTGAAAAA |
| Caspase-8 | TTTCTGCCTACAGGTTCCACT | TGTCCAACTTTCCTTCTCCCA |
| Caspase-9 | CTGTCTACGGCACAGATGGAT | GGGACTCGTCTTCAGGGGAA |
| RELA | TGAACCGAAACTCTGGCAGCTG | CATCAGCTTGCGAAAAGGAGCC |
| TTI1 | TCTGCGATTTACCCTGAAGACC | ATGTCCCCATAAGCTGAGTGC |
| CIP2A | TGCGGCACTTGGAGGTAATTTC | AGCTCTACAAGGCAACTCAAGC |
| PFKL | GCTGGGCGGCACTATCATT | TCAGGTGCGAGTAGGTCCG |

**Figure S1. Schematic of the screening strategy to identify anti-tumor agents from an FDA-approved drug library.**

**Figure S2. PF triggers GSDME-mediated pyroptosis.**

A-B) Representative bright-field images of HeLa cells after treatment with PF at the indicated concentrations for 10 h (A), and with 10 μM PF for the indicated time points (B). C-D) Measurement of LDH release from HeLa cells after treatment with PF at the indicated concentrations and for the indicated time points. E) Representative images of bright field in A375 cells following treatment with 10 μM PF and various inhibitors: 5 μM Fer-1; 10 μM TTM; 10 μM Nec-1s; 10 μM DTT; 10 μM CQ; 40 μM Z-DEVD; 40 μM Z-VAD. F) Representative bright-field images of sgCTRL and sgGSDME HeLa and A375 cells treated with 10 μM PF for 10 h. G) Quantitative analysis of cell death in sgCTRL and sgGSDME HeLa and A375 cells treated with 10 μM PF for 10 h. H) Western blot analysis of Cleaved-caspase1 in HeLa and A375 cells treat with 10 μM PF for the indicated times, and BMDM cells treated with LPS for the indicated times. I) Validation of CASPASE-3, CASPASE-8, CASPASE-9 knockdown efficiency at the mRNA level by quantitative RT-PCR in A375 and HeLa cells. J) Western blot analysis confirms CASPASE-8 and CASPASE-3 knockout in HeLa and A375 cells. K) Measurement of cell viability in siNC, siCASPASE3-, siCASPASE8-, and siCASPASE9-transfected HeLa and A375 cells following treatment with 10 μM PF for 10 h.

**Figure S3.** **PF induces pyroptosis independent of CIP2A and PFKL.**

A) Principal component analysis (PCA) showing the clear separation among samples from the three experimental groups. B) Volcano plot of differentially expressed genes between PF- and DMSO-treated groups. C) Validation of TTI1 knockdown efficiency at the mRNA level by quantitative RT-PCR in A375 cells. D-E) Measurement of cell viability in shCTRL and shTTI1 A375 and HeLa cells after treatment with 10 μM PF for 10 h. F) Validation of CIP2A knockdown efficiency at the mRNA level by quantitative RT-PCR in A375 cells. G) Measurement of cell viability in shCTRL and shCIP2A A375 cells treated with 10 μM PF for 10 h. H) Validation of PFKL knockdown efficiency at the mRNA level by quantitative RT-PCR in A375 cells. I) Measurement of cell viability in shCTRL and shPFKL A375 cells treated with 10 μM PF for 10 h. J) Western blot analysis confirms successful TTI1 overexpression in TTI1 HeLa and A375 cells. K) Western blot analysis of p-p65 levels and GSDME cleavage in A375 Vector and OETTI1 cells following exposure to 10 μM PF for the indicated time. L) Western blot analysis of TTI1 and GSDME expression in the indicated cell lines. M) TTI1 expression in tumor and adjacent normal tissues from the indicated tumor type as sourced from The Human Protein Atlas. (Light color indicates adjacent normal tissue, while dark color indicates tumor tissue).

**Figure S4. PF treatment does not affect mouse body weight or alter the infiltration of CD4⁺ T cells, NK cells, and Tregs.**

A-B) Body weight of NCG (A) and C57 BL/6 mice (B) during treatment with DMSO or PF. C-J) Flow cytometry analysis of intratumoral immune cell subsets: CD8^+^ T cells (C), GZMB^+^ CD8 (D), IFNγ^+^ CD8 (E), CD4^+^ T cells (F-G), NK cells (H), Tregs (I), and dendritic cells (J). K) Analysis of ICD scores in melanoma cells treated with PF for 3 h and 6 h. L) Pathways enrichment analysis of differentially expressed genes in melanoma cells (6 h PF versus DMSO). M) Western blot analysis confirms successful GSDME knockdown in B16F10 cells. N) Body weight of mice bearing subcutaneous B16F10 tumors expressing shCTRL or shGSDME, treated with DMSO or PF.

**Figure S5. PF shows no evidence of weight loss or organ toxicity.**

A) Representative hematoxylin and eosin (H&E) staining of the myocardium, liver, spleen, lung, and kidneys from Braf/Pten-driven spontaneous melanoma mice treated with PF or DMSO. B) Body weight changes in Braf/Pten-driven spontaneous melanoma mice during treatment with DMSO or PF.

**Figure S6. Evaluation of pyroptosis-immune microenvironment interactions in cancer immunotherapy.**

A) Heatmap showing the normalized pyroptosis score and immune infiltration pathway enrichment scores of each sample calculated by single-sample Gene Set Enrichment Analysis (Ribas’ cohort). B-C) TTI1 expression, TNFA Signaling Via NFKB activity, CD8^+^ cell infiltration levels, T cell inflammatory gene expression profile, cytolytic activity, and effector memory T cell infiltration levels in xiangya cohort (B) and Ribas’s cohort (C).

**Figure S7. Single-Cell Profiling of Tumor Ecosystems and Stratification of Malignant Cells by Pyroptosis and Pathway Activity.**

A) t-SNE plot of scRNA-seq clusters from tumor tissues in two datasets (GSE200218 and GSE189889). B) Dot plot showing the specifically expressed genes for the major cell types in two datasets (GSE200218 and GSE189889). C) t-SNE analysis of melanoma scRNA-seq data depicts tumor cell clustering, stratified by pyroptosis sensitivity, TNFA pathway activation status, and TTI1 expression levels in GSE189889 dataset.
